# Fatty acid synthase-mediated lipid droplet formation enhances macrophage killing of *Staphylococcus aureus*

**DOI:** 10.1038/s41419-025-08044-7

**Published:** 2025-10-07

**Authors:** Yanping Wu, Jiaxin Shen, Shenwei Gao, Miao Li, Qingyu Weng, Kua Zheng, Chen Zhu, Zhongnan Qin, Jieyu Li, Jiafei Lou, Songmin Ying, Yinfang Wu, Zhihua Chen, Wen Li

**Affiliations:** Key Laboratory of Respiratory Disease of Zhejiang Province, Department of Respiratory and Critical Care Medicine, Second Affiliated Hospital of Zhejiang University School of Medicine, Hangzhou, 310009 Zhejiang China

**Keywords:** Bacterial infection, Lipid signalling

## Abstract

Macrophages play a critical role in defending against *Staphylococcus aureus* (*S. aureus*), a major human pathogen. Recently, there has been growing interest in the metabolic regulation of macrophage function; however, the specific role of lipid synthesis in macrophage activation remains poorly understood. This study demonstrates that fatty acid synthase (FASN), an enzyme integral to de novo lipogenesis, is significantly upregulated in macrophages during *S. aureus* infection. Notably, *S. aureus* engages in a functional interaction with proteasomes, inhibiting their activity through the PI3K/AKT/mTOR signaling pathway. This interaction results in reduced degradation of FASN, leading to elevated levels of this crucial enzyme. The increased expression of FASN is vital for macrophage-mediated pathogen clearance, as it facilitates the formation of lipid droplets (LDs), which in turn enhance the antimicrobial response against *S. aureus*, partly through the accumulation of the antimicrobial peptide CAMP. In a murine pneumonia model, deficiency of FASN correlates with increased bacterial burden, exacerbated lung inflammation, and a significant reduction in survival rates. Collectively, these findings underscore the essential role of FASN-mediated LD formation in macrophage activation and highlight potential therapeutic targets within the FASN and lipid metabolism pathways for the treatment of *S. aureus* pneumonia.

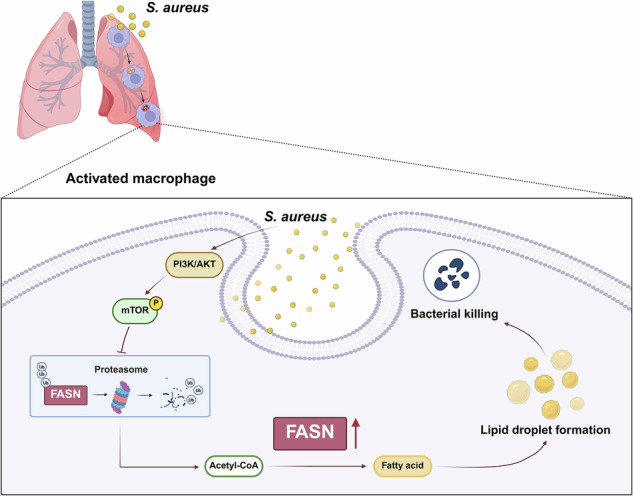

## Introduction

*Staphylococcus aureus* (*S. aureus*) is a common pathogen that causes community-acquired pneumonia (CAP) worldwide. *S. aureus*-induced CAP is often more severe than other CAP types, notable for its severity and substantial clinical implications. Despite representing a relatively small proportion of hospitalized CAP cases—roughly 2–8%—its pronounced clinical presentations and the risk of adverse outcomes make it particularly concerning [1, 2]. The immune response to *S. aureus* infection is complex. Macrophages are fundamental players in the host’s defense against pathogen invasion. During *S. aureus* infection, macrophages are thought to be key for bacterial eradication [3]. Macrophages are responsible for the phagocytic uptake of most invading *S. aureus* and employ a multitude of mechanisms to kill bacteria effectively. Despite this, some *S. aureus* can survive within macrophage phagosomes by evolving an array of strategies to impede macrophage recruitment, phagocytosis, and degrative abilities [4]. Thus, defining how macrophages interact with *S. aureus* will help define an effective innate immune response to *S. aureus* and provide promising new antibacterial strategies.

In recent years, a growing number of studies [5, 6] have highlighted that changes in intracellular metabolic pathways in macrophages could alter their function, which in turn contributes to shaping immune responses. For example, proinflammatory macrophages possess enhanced glycolysis [7]. This is a prerequisite to promote inflammation and fight bacteria as glycolysis inhibitors could reduce macrophage activation. While alterations in glucose metabolism have been well characterized to drive pro-inflammatory phenotype, the interplay between metabolism and immune response during pathogen infection is more complex. Macrophage dysfunction has been identified to be coordinated in part by perturbations in lipid homeostasis [8]. The activation of macrophages leads to an altered abundance of monounsaturated fatty acids [9]. Former reports have also revealed *S. aureus* infection results in a shift of glutamine metabolism and tricarboxylic acid (TCA) cycle [10]. Whereas, how the lipid synthesis pathway changes in *S. aureus*-infected macrophages remains in the process of being elucidated.

Fatty acid synthase (FASN) is a multienzyme protein responsible for de novo lipogenesis [11]. It utilizes acetyl-CoA and malonyl-CoA as substrates to synthesize 16-carbon saturated fatty acid palmitate (C16:0) in NADPH-dependent reaction, during which various chemical steps are catalyzed via respective catalytic domains [12]. Acetyl-CoA is predominantly generated in the mitochondrial matrix by a multitude of catabolic metabolisms and is transported to the cytoplasm via the “citrate-malate-pyruvate shuttle” [13]. The anabolic metabolisms of acetyl-CoA in cytoplasm are sophisticatedly regulated and FASN is considered to be the rate-limiting enzyme of de novo lipogenesis. The initial product palmitate undergoes elongation and desaturation to derive complex lipids. Palmitate is incorporated into triglyceride and phospholipid serving as intracellular lipid storage and membrane structures [14]. Although it has reported that FASN can promote NLRP3 inflammasome activation in macrophages [15], it has not been elucidated the role of FASN in macrophage’s defense against *S. aureus*.

Previous studies have highlighted the pivotal role of lipid droplets (LDs) in the immune response [16]. LDs, once thought to be passive lipid storage organelles [17], are now recognized as dynamic organelles involved in host defense by serving as platforms for the production of antimicrobial peptides and the regulation of lipid-based signaling pathways [18, 19]. For example, LDs in macrophages infected with Mycobacterium tuberculosis have been shown to be a host-driven component of the adaptive immune response [20]. These findings suggest that LDs may serve similar functions during *S. aureus* infection, particularly through their interaction with FASN.

Previous research has provided significant insights into macrophage metabolism and its impact on infection [21, 22]. Building on these findings, we show that pathogenic macrophages display a metabolic phenotype of upregulating FASN protein level in response to *S. aureus*. FASN mediates the formation of LDs, which enable an effective antimicrobial response to *S. aureus* infection but do not affect the phagocytosis function of macrophages. Consistent with in vitro results, myeloid-specific Fasn-deficient (*LysMCre-Fasn*^*f/f*^) mice have a higher bacterial load, causing exacerbated airway infection and reduced survival rate to *S. aureus* pneumonia. Thus, our study identifies FASN as an important metabolic program for macrophage function and highlights its contribution to *S. aureus* pneumonia. Further research into metabolic pathways involved in macrophage activation and bacterial clearance may offer new strategies for treating severe infections and managing inflammatory responses. Continued exploration of lipid metabolism and its impact on immune function could lead to the development of targeted interventions for conditions like *S. aureus* pneumonia.

## Materials and methods

### Mice

C57BL/6 mice were purchased from Shanghai SLAC Laboratory Animal Co. Ltd. (Shanghai, China). LysMCre and *Fasn*^*f/f*^ mice were purchased from the Jackson Laboratory and Cyagen Biosciences (Suzhou, China), respectively. Conditional knockout mice strain-*LysMCre-Fasn*^*f/f*^ mice were generated by crossing LysMCre mice with *Fasn*^*f/f*^ mice. All mice were on the C57BL/6 background. The genotypes of transgenic mice and their control littermates were confirmed by polymerase chain reaction (PCR) analysis of tail snip DNA. Mice were housed in a specific pathogen-free facility at the Laboratory Animal Center of Zhejiang University with a 12-h light/dark cycle and controlled temperature. The mice used for the experiments were 8–12 weeks old and weighed between 23 and 28 g. Mice were randomized into different groups. Male and female mice were sex-matched.

### Cells

Mouse peritoneal macrophages (PMs) were harvested 4 days after thioglycolate (Merck) intraperitoneal injection and cultured in Dulbecco’s Modified Eagle’s Medium (DMEM) supplemented with 10% fetal bovine serum (FBS) and 1% penicillin/streptomycin. Bone marrow cells were collected from tibias and femurs with cold DMEM and were cultured in DMEM supplemented with 10% fetal bovine serum, 10 ng/mL macrophage colony-stimulating factor (M-CSF), and 1% penicillin/streptomycin, to generate bone marrow-derived macrophages (BMDM). THP-1 cells, a human monocytic cell line, were from American Type Culture Collection (#TIB-202) and cultured in RPMI-1640 medium supplemented with 10% FBS and 1% penicillin/streptomycin. For differentiation into macrophage-like cells, THP-1 cells were treated with 100 nM phorbol 12-myristate 13-acetate (PMA) for 48 h. All cells were cultured at 37 °C under an atmosphere containing 5% carbon dioxide. All cell lines were regularly tested for mycoplasma infection.

### *S. aureus* colonization

The *S. aureus* strain was a clinical isolate (multilocus sequence type ST15 and agr type II) with Hld, PSMα, Hla, and PVL+++−. *S. aureus* was grown in Tryptic Soy Broth (TSB) medium at 250 rpm at 37 °C to mid-log phase (optical density at 600 nm of 0.6). Bacteria were collected by centrifugation at 6000 rpm for 10 min and resuspended in sterile phosphate-buffered saline (PBS). Bacteria count was quantified by serial dilutions and plating on TSB plates.

### Antibodies and reagents

Primary antibodies used for immunofluorescence and western blot are as follows: anti-actin beta (ACTB) (ABclonal, #AC037), anti-FASN (Cell Signaling Technology (CST, #3180)), anti-ubiquitin (CST, #3936), anti-phosphorylated mammalian target of rapamycin (p-mTOR) (Abcam, #ab109268), anti-phosphorylated AKT (p-AKT) (Abcam, #ab38449), and anti-cathelicidin antimicrobial peptide (CAMP) (Abcam, #ab318195). Reagents as follows: C75 (#HY-12364), MG-132 (#HY-13259), rapamycin (#HY-10219), ACC inhibitor (CP-640186, #HY-15259), ACLY inhibitor (BMS-303141, #HY-16107), DGAT1 inhibitor (A922500, #HY-10038), and DGAT2 inhibitor (PF-06424439, #HY-108341), all from MedChemExpress; Oleic acid (OA) from Sigma-Aldrich (#O1383); M-CSF from Novoprotein (#CB34); and 4,4-difluoro-1,3,5,7,8-pentamethyl-4-bora-3a,4a-diaza-s-inda-cene (BODIPY 493/503) from Invitrogen (#D3922). Primers for *Fasn*, *Actb*, *Interleukin 6 (Il6)*, and *Interleukin 1 beta (Il1β)* were synthesized by Sangon Biotech. FASN siRNA (#sc-41516) and control siRNA (#sc-37007) were from Santa Cruz Biotechnology.

### In vitro *S. aureus* infection

For in vitro *S. aureus* infection, cells were washed three times with PBS and cultured with DMEM supplemented with 10% FBS without penicillin/streptomycin. *S. aureus* was added at a ratio of 10 to 1 cell (multiplicity of infection (MOI) = 10), except experiments indicated.

### siRNA transfection

When BMDM reached 60–80% confluence in 12-well plates, siRNA transfection was performed. For each well, 90 nM FASN siRNA or control siRNA (Santa Cruz Biotechnology) was diluted in 100 μL of Opti-MEM (Gibco, #31985070), mixed with 6 μL of Lipofectamine RNAiMAX (Thermo Fisher Scientific, #13778150), and incubated for 15 min at room temperature to allow complex formation. The mixture was then added dropwise to the cells with gentle swirling to ensure even distribution. Cells were incubated for 24 h before subsequent treatments.

### Western blot analysis

Cells were lysed in RIPA lysis buffer (Beyotime, #P0013B) containing cOmplete™ Protease Inhibitor (Roche, #04693116001) and PhosSTOP™ (Roche, #04906845001). The collected samples were ultrasonicated and centrifugated at 12,000 rpm for 5 min. Protein concentrations in supernatants were measured by BCA Protein Assay Kit (ThermoFisher, #23225) and equivalent amounts of protein from each sample were added to 5× loading buffer (0.25 M Tris-Cl, 50% glycerol, 10% SDS, 2% β-mercaptoethanol, 0.25% bromophenol blue, pH 6.8). After being heated at 100 °C for 10 min, proteins were loaded and separated on 6–10% SDS-PAGE gels. Then, proteins were blotted onto PVDF membranes (Millipore, #88518). Membranes were blocked with 5% non-fat milk in TBST for 1 h at room temperature (RT) and incubated with primary antibody at 4 °C overnight. Membranes were incubated with secondary antibodies for 1 h at RT and scanned using a western blot detection system (Odyssey, Li-COR Bioscience).

### Ubiquitination level of endogenous FASN protein

Cells were washed three times in cold PBS and lysed in lysis buffer (ABclonal, # RM02998) supplemented with cOmplete™ Protease Inhibitor (Roche) and PhosSTOP™ (Roche). Cell lysates were centrifugated at 12,000 rpm for 10 min at 4 °C. Cleared cell lysates were immunoprecipitated with the anti-FASN antibody (CST) at 4 °C overnight, which was pre-adsorbed on Protein G Magnetic Beads (Bio-Rad). Beads were washed three times with lysis buffer and protein samples were eluted with 1 × SDS loading buffer via heated at 100 °C for 10 min. Samples were analyzed by western blotting with the primary antibody of anti-FASN (CST) and anti-ubiquitin (CST).

### RNA isolation and reverse transcription quantitative PCR (qPCR)

RNA was extracted using Trizol (Invitrogen, #15596018CN) following the manufacturer’s protocol. Reverse transcription of 1 μg of total RNA was carried out using the PrimeScript™ RT reagent Kit (Takara, #RR037A), and cDNA was synthesized. The expression of mouse *Actb* and *Fasn* were measured by Real-time qPCR on a StepOnePlus PCR system (Applied Biosystems). Data were calculated using the 2^−ΔΔCt^ method and normalized to *Actb* expression. The mouse primers used for qPCR are as follows: *Actb* (*actin beta*, amplicon size 245 bp, forward GTGACGTTGACATCCGTAAAGA, reverse GCCGGACTCATCGTACTCC); *Fasn* (*Fatty acid synthase*, amplicon size 99 bp, forward AGAGATCCCGAGACGCTTCT, reverse GCTTGGTCCTTTGAAGTCGAAGA); *Il6* (*Interleukin 6*, amplicon size 131 bp, forward CTGCAAGAGACTTCCATCCAG, reverse AGTGGTATAGACAGGTCTGTTGG); *Il1β* (*Interleukin 1 beta*, amplicon size 116 bp, forward GAAATGCCACCTTTTGACAGTG, reverse TGGATGCTCTCATCAGGACAG).

### Immunofluorescence analysis

Cells were seeded at a density of 2 × 10^5^ cells per well in 12-well plates and treated as indicated. After washing twice with PBS, the cells were fixed in 4% paraformaldehyde in PBS for 15 min at RT and washed three times with PBS. Then, cells were permeabilized with 0.5% Triton X-100 and blocked with 5% bovine serum albumin in PBS. For immunostaining, cells were incubated with primary antibodies overnight at 4 °C, followed by incubation with secondary antibodies conjugated to Alexa Fluor 488 or 555 (Life Technologies) for 1 h at RT in the dark. DAPI was used to stain the nuclei. For lipid droplet staining, cells were incubated with a 2 µM Bodipy 493/503 at 37 °C for 15 min in the dark, followed by DAPI staining. Images were visualized using a high-resolution laser Confocal Microscope (Olympus IX83-FV3000-OSR) and processed with FV31S-SW software (Olympus).

### ELISA

Cytokine levels in the supernatant of bronchoalveolar lavage fluid (BALF) were measured using Mouse interleukin 6 (IL6) DuoSet ELISA (R&D Systems, #DY406) and Mouse CXC motif chemokine ligand 1 (CXCL1) DuoSet ELISA Kit (R&D Systems, # DY275) according to the manufacturer’s instructions. Absorbances at 450 nm were measured on a VersaMax microplate reader (Molecular Devices). Cytokine concentrations were calculated by extrapolating absorbance values from standard curves where known concentrations were plotted against absorbance.

### Flow cytometric analysis

Cells were centrifuged at 400 × *g* for 5 min at 4 °C and resuspended in 50 µL of PBS. To evaluate cellular lipid level, cells were suspended in Bodipy 493/503 and protected from light for 30 min at 37 °C. Finally, cells were assayed using a FACSCalibur flow cytometer (Cytoflex) and analyzed with FlowJo software (version 10; Tree Star).

### Transmission electron microscopy

Macrophages were fixed in 2.5% glutaraldehyde for 1 h at RT, followed by overnight fixation at 4 °C. After three washes with PBS, the cells were post-fixed with 1% osmium tetroxide in PBS for 1.5 h and subsequently stained with 2% uranyl acetate. The samples were dehydrated through a graded ethanol series and embedded in resin. Ultrathin sections were cut, stained with 1% uranyl acetate and 0.4% lead citrate, and imaged using a transmission electron microscope (Tecnai G2 Spirit 120 kV) at the Centre of Cryo-Electron Microscopy, Zhejiang University.

### Proteasome activity detection

The proteasome activity of cells was detected by the Proteasome 20S Activity Assay Kit (Sigma-Aldrich, #MAK172) according to the manufacturer’s instructions. The fluorometric signals were detected on SpectraMax® M5/M5e Multimode Plate Reader (Molecular Devices).

### LD purification

LD purification was performed by Lipid Droplet Isolation Kit (Cell Biolabs, #MET-5011). Briefly, cells were incubated with 120 µM OA for 12 h to induce LDs which were then resuspended pellet thoroughly with 200 µL of Reagent A. They were incubated on ice for 10 min. Then 800 µL of Reagent B was added and mixed well. Cells were homogenized by passing them five times through a one-inch 27 gauge needle attached to a 3 mL syringe. Reagent B was loaded on top of the homogenate. Ultracentrifugation with 18,000 × *g* at 4 °C was performed for 3 h so that the LD fraction would be at the top of the tube.

### LD proteomic analysis

LDs were isolated from macrophages treated with OA or OA plus *S. aureus*, and subjected to protein extraction using 8 M urea supplemented with protease inhibitors. Proteins were reduced with 10 mM TCEP, alkylated with 25 mM CAA, and digested overnight with trypsin (50:1, w/w) in 10 mM TEAB at 37 °C. Peptides were acidified with formic acid, desalted using C18 columns, eluted with 70% acetonitrile, lyophilized, and stored at –80 °C. Peptides were analyzed on a Q Exactive HF-X mass spectrometer (Thermo Fisher Scientific) using a C18 column and data-dependent acquisition. MS data were processed with Proteome Discoverer (v2.4) and searched against the Mus musculus UniProt database using Sequest HT. Search parameters included trypsin digestion (≤2 missed cleavages), 15ppm precursor tolerance, 0.02 Da fragment tolerance, fixed carbamidomethylation (C), and variable oxidation (M). Peptide and protein FDRs were set at <1%. Protein abundance was expressed as scaled normalized abundance. Relative abundance profiles across samples were visualized using hierarchical clustering heatmaps generated in R, with color intensity representing scaled abundance levels. The proteomic data have been deposited in the iProX repository under accession number PXD064032.

### Bacterial load measurement

For supernatants of cell culture, supernatants were centrifugation at 6000 rpm for 10 min at 4 °C, and bacterial pellets were resuspended in sterile PBS. After serially diluted in PBS, samples were plated on TSB plates and incubated at 37 °C overnight. The colony-forming units (CFUs) were enumerated.

For lung tissues, the left lungs were homogenized in sterile PBS. Homogenates were serially diluted in PBS and plated on TSB plates. After incubating at 37 °C overnight, CFU was enumerated.

### Phagocytosis assay

The phagocytosis rate was detected by a phagocytosis assay kit (Red Zymosan) (Abcam, # ab234054). Briefly, cells were washed three times with PBS and incubated with Red Zymosan at 37 °C for 1 h. Cells were harvested by centrifugation at 400 × *g* for 5 min and resuspended in PBS. Fluorescence signals were acquired on a CytoFlex analyzer (Beckman Coulter) at Ex/Em 540/570 nm and data were analyzed using FlowJo software (version 10; Tree Star).

### Scratch wound healing assay

For evaluating macrophage migration, scratch wound healing assay was employed. Confluent BMDM monolayers in 6-well plates were scratched using a 200 µL pipette tip. After washing with PBS, cells were treated with DMSO and C75 in DMEM with 2% FBS. Images were captured at 0 and 24 h, and scratch length was quantified using ImageJ software.

### Cell adhesion assay

For cell adhesion assays, THP-1 cells were differentiated with 100 nM PMA for 48 h. The wells of a 24-well plate were coated with 0.1% gelatin and incubated overnight. Cells (1 × 10^5^ per well) were plated and allowed to adhere for 1 h at 37 °C. After washing with PBS, adherent cells were fixed in 4% paraformaldehyde and stained with 0.1% crystal violet.

### Murine bacterial infection

For murine bacterial infection, age-matched 8-10 week-old mice, weighing between 23 and 28 g, were studied, and 5 × 10^6^ *S. aureus* in 50 µL sterile saline was intratracheally inoculated per mouse. Control animals were intratracheally infused with 50 µL sterile saline only. For lethal infections in the survival curve analysis experiment, 1 × 10^8^ *S. aureus* in 50 µL sterile saline was intratracheally inoculated per mouse. Mice were monitored twice daily for mortality.

### Bronchoalveolar lavage

Twenty-four hrs after bacterial lung infection, mice were sacrificed for analysis. BALF was collected by instilling 1 mL PBS into the left lung through the trachea. The total BAL cells were counted, and the rest of the BALF was centrifuged at 400 × *g* for 15 min at 4 °C. Then the cell types in the BALF were identified based on Wright-Giemsa staining. Cytokine levels in supernatants were determined by ELISA (R&D Systems) as described previously.

### Lung histology

Lung tissue was fixed in a 10% formalin solution and embedded in paraffin. Lung sections were cut and stained with hematoxylin and eosin (H&E) or subjected to gram stain by the Histopathology Core Platform of Zhejiang University School of Medicine. Images of lung sections were visualized on an Olympus BX53 microscopy. The inflammation score was assessed on a subjective scale of 0-3 based on published guidelines [23].

### RNA sequencing data analysis

RNA-seq data were retrieved from the Gene Expression Omnibus (GEO) database (GSE272198) [24]. Differential expression analysis was performed using DESeq2 (V1.42.1), with upregulated genes identified based on an adjusted *P*-value < 0.05 and log_2_ fold change > 0. These upregulated genes were subsequently subjected to Kyoto Encyclopedia of Genes and Genomes (KEGG) pathway enrichment analysis using the ClusterProfiler package (V4.12.6) in R. The top 20 enriched KEGG pathways were visualized using a bubble chart to highlight the most relevant biological processes. In this chart, the x-axis represents the gene ratio, calculated as the number of upregulated genes mapped to a given pathway divided by the total number of upregulated genes analyzed; the bubble size (“count”) reflects the number of upregulated genes enriched in each pathway; and the y-axis displays the statistical significance of pathway enrichment, expressed as the negative logarithm of the adjusted *p*-value [−log_10_(*p* value)], where a higher value indicates stronger enrichment.

### Statistics

Sample sizes were determined based on preliminary experiments and previous publications. No samples were excluded from the analysis. Animals were randomly assigned to experimental groups where applicable. The investigator was blinded to group allocation during histological assessment, but blinding was not applied for other analyses. Data were tested for normality and presented as the mean ± standard error of the mean (SEM). For comparisons between two groups, an unpaired two-tailed Student’s *t* test was used. Comparisons among multiple groups were conducted using one-way ANOVA, followed by Tukey’s post-hoc test for multiple comparisons. For experiments involving two independent variables, two-way ANOVA was performed with Sidak’s post-hoc test. Kaplan–Meier survival curves were generated, and survival differences between groups were evaluated using the log-rank (Mantel–Cox) test. All statistical analyses were performed using GraphPad Prism 8 software. Differences were considered statistically significant when the *P* value was less than 0.05. The raw experimental data for all comparisons have been provided in Supplementary Table 1.

## Results

### Macrophage de novo fatty acid synthesis is induced during *S. aureus* infection

**Fig. 1:**
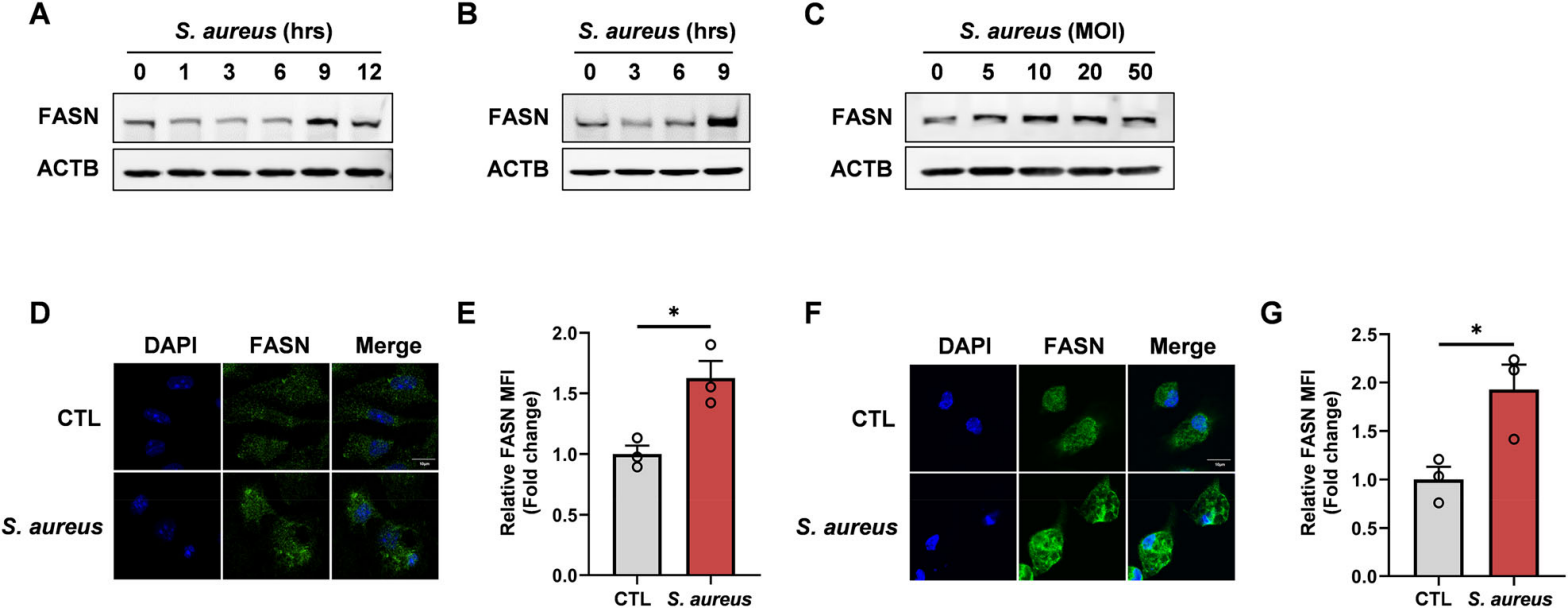
*S. aureus* stimulation promotes the de novo fatty acid synthesis pathway in macrophages. Macrophage de novo fatty acid synthesis is induced during *S. aureus* infection. Western blot analysis of FASN in BMDM (**A**) and PMs (**B**) stimulated with *S. aureus* (MOI = 10) for the indicated duration before harvest. **C** Western blot analysis of FASN in BMDM stimulated with indicated MOIs of *S. aureus* for 9 h. The protein level of FASN was determined by immunofluorescence in BMDM (**D**, **E**) and PMs (**F**, **G**) after exposure to *S. aureus* (MOI = 10) for 9 h. Representative immunofluorescence images (**D**, **F**) and statistical analysis of relative protein MFI (**E**, **G**). Scale bar, 10 μm. Green, FASN; blue, DAPI. All data are shown as mean ± SEM and analyzed using an unpaired two-tailed Student’s *t* test. *P < 0.05.

We first detected FASN protein levels to analyze whether *S. aureus* stimulus influenced fatty acid synthesis in macrophages. Western blotting revealed a significant elevation of FASN in macrophages exposed to *S. aureus*, with a time-dependent increase starting to significantly accumulate at 9 h post-infection, and with the most significant increase occurring at an MOI of 10 (Fig. 1A–C). Immunofluorescence staining further confirmed these results, showing a marked increase in FASN levels in macrophages infected with *S. aureus* (Fig. 1D–G). These data indicate that *S. aureus* stimulates the upregulation of FASN protein in macrophages, potentially contributing to the host immune response.

### *S. aureus* disturbs FASN degradation via the PI3K/AKT/mTOR pathway by regulating ubiquitin-proteasome system (UPS) activity

**Fig. 2:**
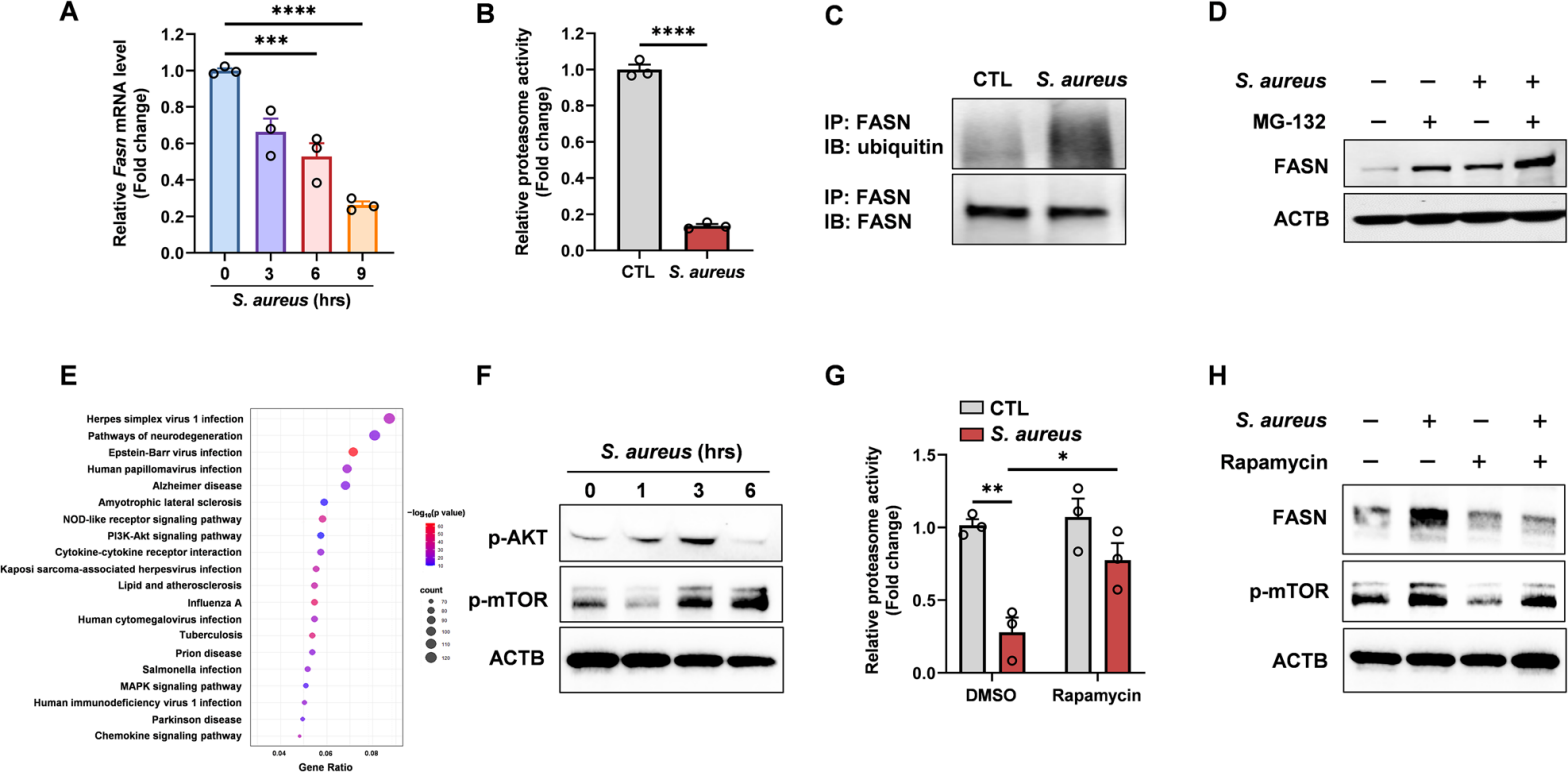
*S. aureus* disrupts FASN degradation through the PI3K/AKT/mTOR pathway by regulating UPS activity. **A** qPCR analysis of FASN in BMDM stimulated with *S. aureus* (MOI = 10) at the indicated times. **B** Proteasome activity was measured in BMDM stimulated with *S. aureus* (MOI = 10) for 9 h. **C** The ubiquitination level of FASN was determined by IP experiments in BMDM treated with *S. aureus* (MOI = 10) for 9 h. **D** Effects of MG-132 (100 nM) on the protein level of FASN treated with *S. aureus* (MOI = 10) for 9 h. **E** Top 20 KEGG pathways enriched in genes upregulated in BMDM infected with *S. aureus*. **F** Western blot analysis of p-AKT and p-mTOR in BMDM infected with *S. aureus* (MOI = 10) for the indicated duration before harvest. **G** Proteasome activity was measured in BMDM treated with Rapamycin (25 nM) and infected with *S. aureus* (MOI = 10) for 9 h. **H** Western blot analysis of FASN following mTOR inhibition in BMDM infected with *S. aureus* (MOI = 10) for 9 h. All data are shown as mean ± SEM, analyzed using an unpaired two-tailed Student’s *t* test (**B**), one-way ANOVA (**A**), or two-way ANOVA (**G**). *P < 0.05, **P < 0.01, ***P < 0.001, ****P < 0.0001.

To clarify the mechanism underlying the elevated protein expression of FASN during *S. aureus* infection, we first measured its transcription level. Surprisingly, qPCR analysis revealed that FASN mRNA levels were downregulated upon *S. aureus* infection (Fig. 2A). Previous studies have found that FASN could be degraded via the UPS in tumor cells [25]. This led us to hypothesize that post-translational regulation, specifically through the UPS, could be involved. Thus, we investigated whether the degradation pathway of FASN was influenced by *S. aureus* infection. UPS contains two parts—firstly a series of enzymes contributing to protein ubiquitination, and secondly a complex called proteasome responsible for degrading protein into peptides [26]. Thus, we detected these two aspects individually. Remarkably, proteasome activity in macrophages is dramatically downregulated after *S. aureus* interference (Fig. 2B), while the ubiquitination level of FASN protein is upregulated (Fig. 2C). This suggests that *S. aureus* suppresses proteasomal degradation of FASN, leading to its accumulation. We also used MG-132, a proteasome inhibitor, to confirm this mechanism. Co-treatment with MG-132 and *S. aureus* further amplified the FASN accumulation (Fig. 2D). This raises the possibility that *S. aureus* may also regulate FASN expression through proteasome-independent pathways. Alternatively, the additive effect may reflect differences in the extent of proteasome inhibition, with MG132 exerting stronger suppression than *S. aureus*. Based on this, we propose that *S. aureus* may enhance FASN expression at least in part by partially attenuating proteasomal degradation, possibly in conjunction with additional regulatory mechanisms.

To further elucidate the molecular mechanism by which *S. aureus* regulates proteasome activity, we conducted a transcriptome analysis using data retrieved from the GEO database (GSE272198) [24]. KEGG pathway enrichment analysis based on genes significantly upregulated after *S. aureus* infection revealed prominent enrichment of the phosphatidylinositol 3-kinase (PI3K)/AKT, nucleotide-binding oligomerization domain (NOD)-like receptor, and mitogen-activated protein kinase (MAPK) signaling pathways in infected macrophages (Fig. 2E). Previous studies have shown that the PI3K/AKT pathway plays a crucial role in the regulation of proteasome activity. Notably, mTOR, a well-recognized downstream target of PI3K/AKT, is considered a central regulator of proteasome activity [27, 28]. This led us to focus our subsequent research on the PI3K/AKT/mTOR pathway to elucidate its role in the macrophage response to *S. aureus* infection. Results showed that the phosphorylation levels of AKT and mTOR were significantly upregulated following *S. aureus* intervention (Fig. 2F), confirming the activation of the PI3K/AKT/mTOR pathway. Furthermore, mTOR inhibition by rapamycin significantly restored proteasome activity in *S. aureus*-infected macrophages (Fig. 2G). Consistently, rapamycin treatment reduced FASN protein levels in *S. aureus*-infected macrophages, suggesting that mTOR activation contributes to FASN stabilization by suppressing proteasome function (Fig. 2H). However, as rapamycin is also known to inhibit protein translation, we sought to determine whether the observed decrease in FASN was due to restored proteasome activity or reduced protein synthesis. We treated *S. aureus*-infected macrophages with MG132, rapamycin, or both. Western blot analysis showed that MG132 markedly increased FASN levels, and this increase was not reversed by co-treatment with rapamycin (Supplementary Fig. S1). These results indicate that the effect of rapamycin on FASN is primarily mediated through reactivation of proteasomal degradation rather than inhibition of protein translation. In conclusion, *S. aureus* interacts with macrophages through the PI3K/AKT/mTOR pathway to regulate proteasome activity, thereby influencing FASN degradation.

### *S. aureus* killing by macrophages is dependent on FASN

**Fig. 3:**
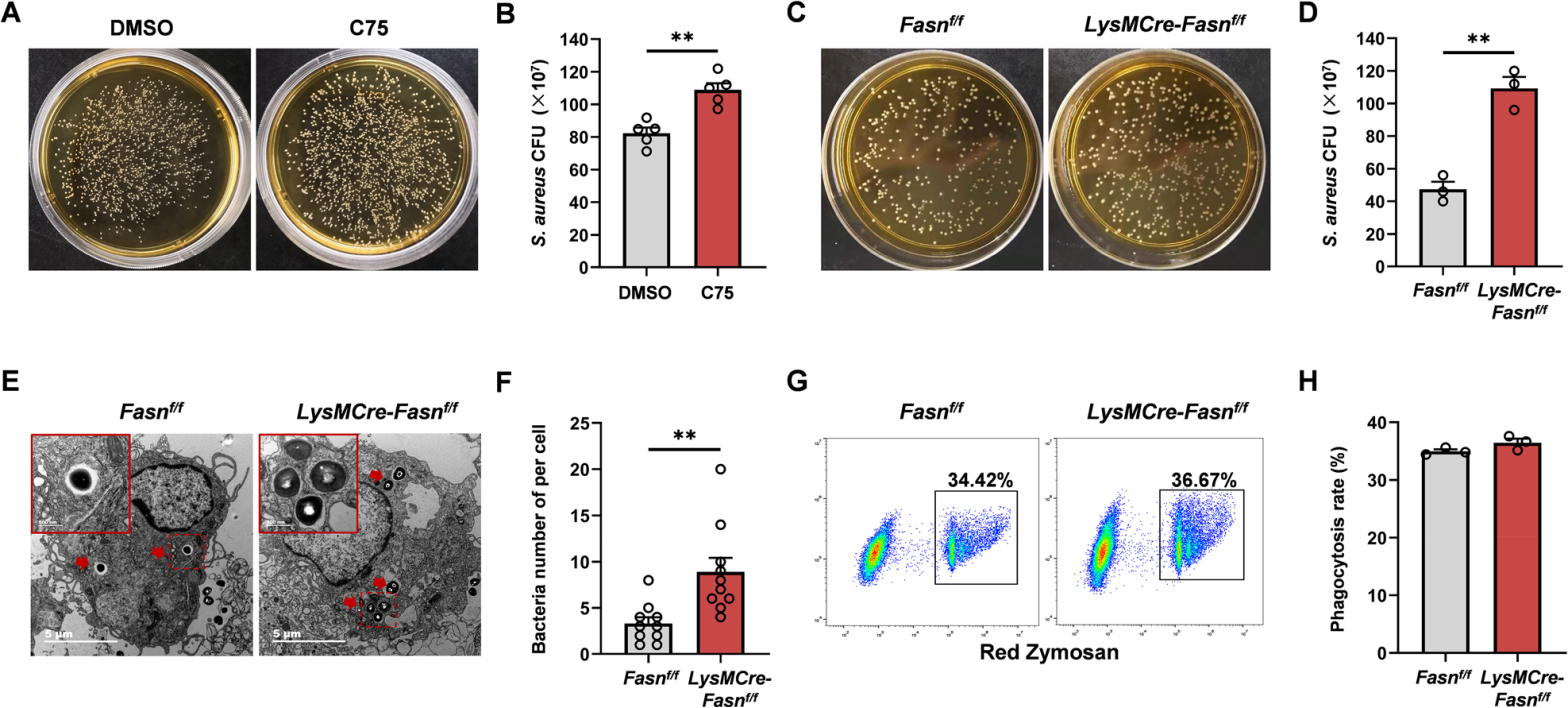
*S. aureus* killing by macrophages is dependent on FASN. Representative images of CFU (**A**) and measurement of bacterial colony number (**B**) in BMDM culture supernatants at 9 h post-*S. aureus* stimulation in the presence or absence of 25 µM C75 (FASN inhibitor). **C**, **D** BMDM isolated from *Fasn*^*f/f*^ and *LysMCre-Fasn*^*f/f*^ mice were cultured with *S. aureus* for 9 h. Representative images of CFU (**C**) and measurement of bacterial colony number (**D**) in cell culture supernatants. **E**, **F** Representative transmission electron microscopy images and statistical analysis of BMDM from *Fasn*^*f/f*^ and *LysMCre-Fasn*^*f/f*^ mice treated with *S. aureus* for 9 h. Red arrowheads point to intracellular bacteria. Scale bar, 5 μm. **G**, **H** BMDM from *Fasn*^*f/f*^ and *LysMCre-Fasn*^*f/f*^ mice was incubated with Zymosan particles pre-labeled with a red dye and subsequently analyzed by flow cytometry. Representative flow cytometry images (**G**) and quantification of BMDM phagocytosis (**H**). All data are shown as mean ± SEM and analyzed using an unpaired two-tailed Student’s *t* test. **P < 0.01.

Macrophages are professional phagocytes equipped with an impressive armamentarium of antimicrobial effectors [29]. In the early phase of infection, macrophages are predominantly activated to phagocytose and kill the invading bacteria. To investigate the role of FASN in macrophage activation, we first set out to determine the influence of FASN on the bactericidal capacity of macrophages. It was found that C75 treatment, a FASN inhibitor, increased the bacteria load of *S. aureus* in culture supernatants (Fig. 3A, B). Additionally, macrophages from *LysMCre-Fasn*^*f/f*^ mice were obtained to rule out the off-target influence of C75. Knockdown of FASN also got a similar result with C75 (Fig. 3C, D). To verify the CFU results, we identify the bacteria-containing cells by a transmission electron microscope. Consistently, our results also showed that intracellular bacteria in FASN-knockdown macrophages were enhanced (Fig. 3E, F). We further set out to determine if bacterial killing was due to the difference in phagocytosis. The phagocytosis assay using zymosan bioparticles showed that FASN knockdown did not disturb the ability for particle engulfment of macrophages (Fig. 3G, H). Taken together, these results suggested that FASN could contribute to increasing the bactericidal activity of macrophages and reducing bacterial burdens, without affecting their phagocytic capacity.

To gain a more comprehensive understanding of the immune response of macrophages in the context of *S. aureus* infection, we extended our investigation beyond antibacterial activity to include assessments of macrophage migration, adhesion, and activation. Our findings revealed that FASN inhibition did not have a significant impact on macrophage migration and adhesion (Supplementary Fig. S2A–D); however, it markedly impaired activation, as evidenced by a significant downregulation of the pro-inflammatory cytokines IL6 and IL1β (Supplementary Fig. S2E–H). This finding corroborates our previous observation that FASN is integral to macrophage bactericidal activity. The proper activation of macrophages is essential for effective bacterial clearance [30]. The impairment in macrophage activation likely contributes to the diminished bactericidal function observed.

### LDs are highly abundant in macrophage response to *S. aureus* infection

**Fig. 4:**
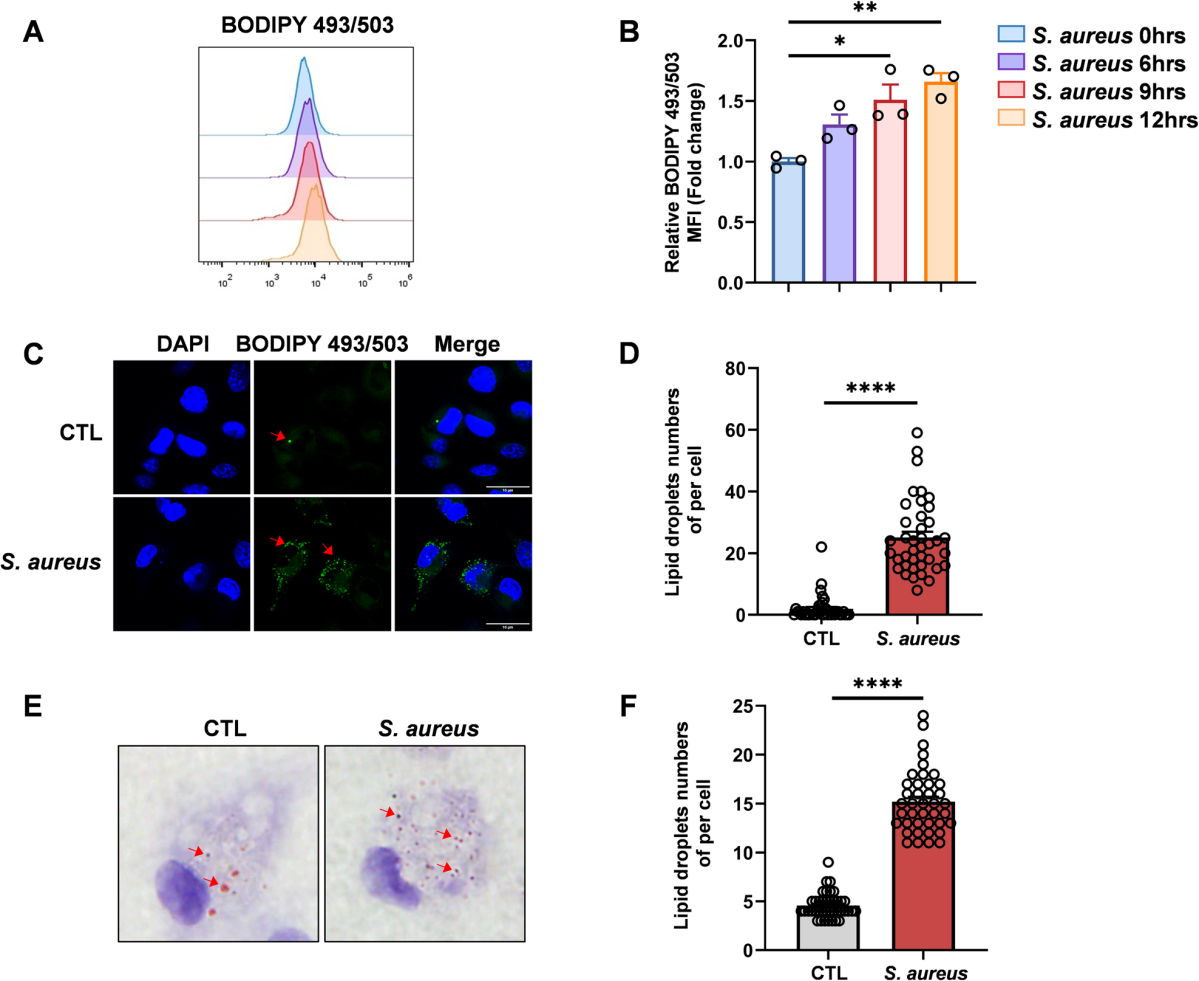
LDs are highly abundant in macrophage response to *S. aureus* infection. Representative histograms (**A**) and MFI values (**B**) of BODIPY 493/503 staining in cultured BMDM. Representative immunofluorescence images (**C**) and quantification of the number of LDs per cell (**D**). Red arrows indicate LDs. Scale bar, 10 μm. Green, BODIPY 493/503; blue, DAPI. Representative oil red O-stained images (**E**) and quantification of the number of LDs per cell (**F**). Red arrows indicate LDs. All data are shown as mean ± SEM, analyzed using one-way ANOVA (**B**) or an unpaired two-tailed Student’s *t* test (**D**, **F**). *P < 0.05, **P < 0.01, ****P < 0.0001.

Our data indicated that FASN was required for the *S. aureus* killing of macrophages, but the underlying mechanism remained unknown. FASN is a well-established regulator of de novo fatty acid synthesis, which is important for the intracellular lipid pool. LDs are the hub for lipid storage that dynamically regulates lipids and energy homeostasis in the cell [31], which is related to FASN-mediated anabolism. Thus, we assessed the potential role of FASN in LD metabolism during *S. aureus* infection. To confirm that we stained macrophage with the neutral lipid dye BODIPY 493/503 to quantify neutral lipids. Consistent with increased FASN protein level, *S. aureus*-infection displayed increased staining with BODIPY 493/503, indicative of markedly increased LD formation (Fig. 4A–D). Oil red O staining, which visualized the morphology of lipid droplets, also showed that *S. aureus*-stimulated macrophages contained more amounts of lipid droplets despite decreased size (Fig. 4E, F). Together, these data indicate that *S. aureus* imprints a metabolic program in macrophages characterized by increased expression of FASN and formation of LDs.

### FASN-dependent LD formation contributes to the killing of *S. aureus*

**Fig. 5:**
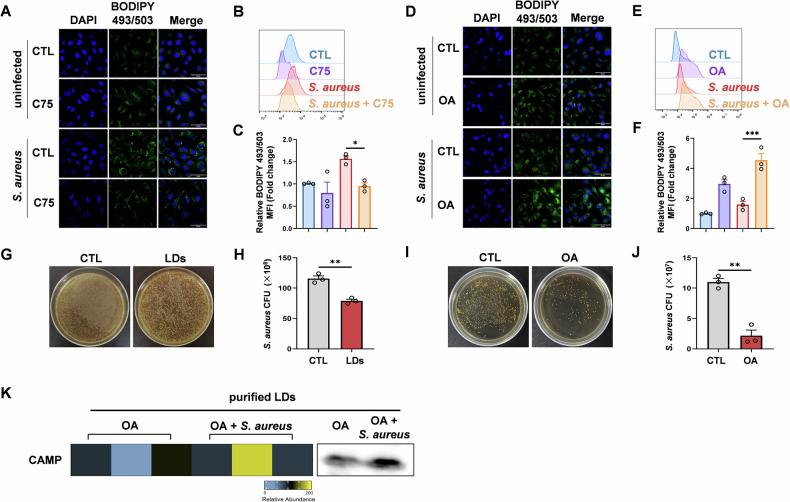
FASN-dependent LD formation contributes to the killing of *S. aureus.* **A**–**C** Treatment with 25 µM C75 in BMDM exposed to *S. aureus* for 9 h, as determined by immunofluorescence and flow cytometry. Representative immunofluorescence images (**A**), representative histograms (**B**), and MFI values (**C**) of BODIPY 493/503 staining in cultured BMDM. Scale bar, 20 μm. Green, BODIPY 493/503; blue, DAPI. **D**–**F** Control and OA-loaded (120 µM) BMDM were infected with *S. aureus* for 9 h. Representative immunofluorescence images (**D**), representative histograms (**E**), and MFI values (**F**) of BODIPY 493/503 staining in cultured BMDM. Scale bar, 20 μm. Green, BODIPY 493/503; blue, DAPI. **G**, **H** LDs were purified from BMDM pre-treated with 120 µM OA for 12 h. *S. aureus* was incubated for 24 h in a standard medium or medium supplemented with purified LDs. Representative images of CFU (**G**) and measurement of bacterial colony number (**H**). **I**, **J** OA-treated or control BMDM were infected with *S. aureus* for 9 h. Representative images of CFU (**I**) and measurement of bacterial colony number (**J**). **K** LDs were isolated from macrophages treated with OA or OA plus *S. aureus*. Mass spectrometry and Western blot analysis of CAMP. All data are shown as mean ± SEM, analyzed using two-way ANOVA (**C**, **F**) or an unpaired two-tailed Student’s *t* test (**H**, **J**). *P < 0.05, **P < 0.01, ***P < 0.001.

The formation of LDs is considered an innate immune hub integrating cell metabolism and host defense [18]. Here, FASN, an enzyme that is responsible for de novo fatty acid synthesis, is important for the intracellular lipid pool and crucial in the process of LD formation. To confirm that FASN is indeed essential in LD formation during *S. aureus* infection, we used C75 to inhibit FASN and observed the following changes in LDs. C75 treatment significantly downregulated the formation of LDs as assessed by confocal microscopy (Fig. 5A). In addition, attenuated LDs were also observed in C75-treated macrophages as assessed by flow cytometry (Fig. 5B, C). To validate the requirement of FASN using a genetic approach, we performed siRNA-mediated knockdown of FASN in macrophages. Immunofluorescence analysis showed that FASN deficiency significantly impaired LD formation after *S. aureus* infection. In line with the loss of LDs, macrophages transfected with FASN siRNA also exhibited diminished bacterial clearance, indicating a functional consequence of FASN deficiency (Supplementary Fig. S3A–C). Efficient knockdown of FASN was confirmed at both mRNA and protein levels (Supplementary Fig. S3D, E). Our results suggested that infection of *S. aureus* strongly upregulated key enzyme-FASN involved in lipid synthesis and LD formation.

To further assess the importance of lipid anabolism in activating macrophage responses to *S. aureus*, we incubated macrophages with OA, the major fatty acid component of LDs. LD accumulation in *S. aureus*-treated macrophages was promoted by incubation with OA, a fatty acid efficiently esterified into LDs (Fig. 5D–F). To test the importance of LD formation for the functionality of macrophages in an inflamed microenvironment, LDs were purified and incubated with *S. aureus*. Bacterial viability was estimated from the resulting CFUs. Bacterial growth was effectively reduced after incubation with LDs (Fig. 5G, H). Building on these observations, we next evaluated whether enhanced LD abundance could improve bacterial control. Indeed, OA-treated macrophages exhibited significantly reduced *S. aureus* loads compared to controls (Fig. 5I, J), indicating that increased LD formation enhances the antimicrobial capacity of macrophages.

To further examine the contribution of the lipid synthesis pathway to LD formation and antibacterial activity, we pharmacologically inhibited key enzymes involved in de novo lipogenesis and triglyceride synthesis, including ACC, ACLY, DGAT1, and DGAT2. Immunofluorescence analysis showed that inhibition of any of these enzymes significantly reduced LD accumulation following *S. aureus* infection. Consistently, CFU assays revealed that impaired LD formation due to enzymatic inhibition led to diminished bacterial clearance by macrophages (Supplementary Fig. S3F–H). These results highlight the essential role of intact lipid metabolic flux and LD biogenesis in supporting macrophage antimicrobial function.

To investigate how LDs exert their antimicrobial effects at the molecular level, we performed mass spectrometry on LD fractions isolated from macrophages treated with OA or OA plus *S. aureus*. Proteomic analysis revealed an enrichment of CAMP, a broad-spectrum antimicrobial peptide, on LDs following infection. This result is consistent with a previous study by Bosch et al. [18], which demonstrated that LD-localized CAMP enhances host defense against various bacterial pathogens, including methicillin-resistant *S. aureus* (MRSA). Western blot analysis further confirmed elevated CAMP protein levels in LDs upon *S. aureus* infection (Fig. 5K). Together, these findings support a model in which LDs act as immune effector organelles that concentrate antimicrobial proteins such as CAMP, thereby promoting bacterial clearance. Thus, FASN expression in macrophages mediates the formation of LDs, which enable an effective antimicrobial response to *S. aureus* infection.

### FASN contributes to reducing lung bacterial burden and protecting the host from *S. aureus* pneumonia

**Fig. 6:**
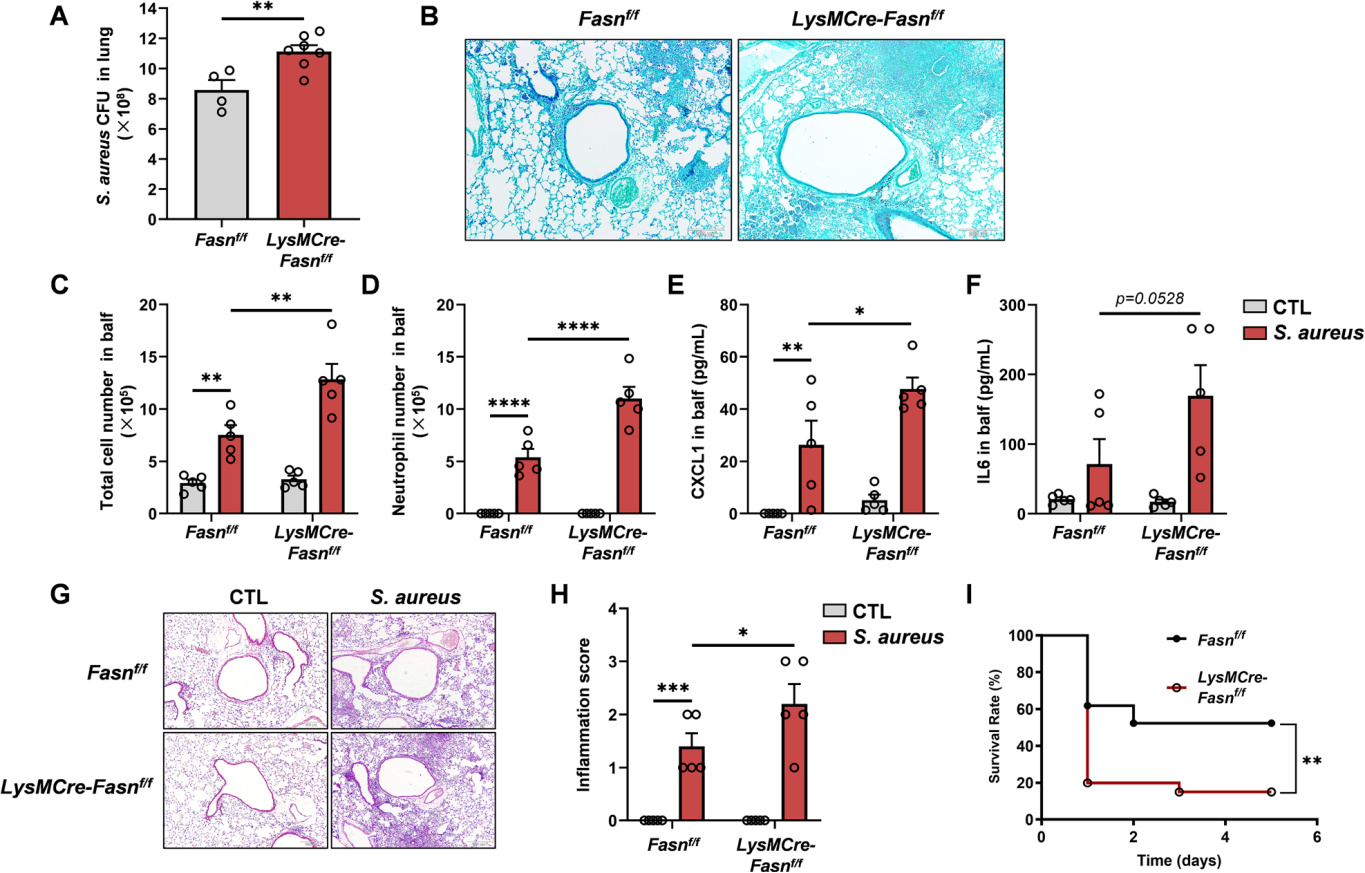
FASN contributes to reducing lung bacterial burden and protecting the host from *S. aureus* pneumonia. **A** Bacterial burden was determined by CFU from *Fasn*^*f/f*^ and *LysMCre-Fasn*^*f/f*^ mouse lungs 24 h after *S. aureus* infection. **B** Gram staining of lung tissues in *Fasn*^*f/f*^ and *LysMCre-Fasn*^*f/f*^ mice 24 h after *S. aureus* infection. Scale bar, 200 μm. **C** Total BALF cell counts. **D** BALF neutrophil counts. **E**, **F** Cytokine levels in BALF were determined by ELISA. **G** Representative H&E-stained lung sections. **H** Inflammation scores. Scale bar, 200 μm. **I** The survival rate of mice after modeling. All data are shown as mean ± SEM, analyzed using an unpaired two-tailed Student’s *t* test (**A**), two-way ANOVA (**C**–**F**, **H**), or the log-rank (Mantel–Cox) test (**I**). *P < 0.05, **P < 0.01, ***P < 0.001, ****P < 0.0001.

An increasing number of studies have suggested that an abundance of proinflammatory macrophages have direct roles in the outcome of *S. aureus* infection [32, 33]. Macrophage lipid synthesis may benefit the host during *S. aureus* pneumonia by enhancing bacterial clearance. We hypothesized that FASN would protect the lung from inflammation and tissue damage during *S. aureus* pneumonia. To determine this hypothesis, we established *LysMCre-Fasn*^*f/f*^ mice to undergo *S. aureus* lung infection. Similar to in vitro experiments, lung bacterial burden was increased in *LysMCre-Fasn*^*f/f*^ mice (Fig. 6A). Gram staining assay also revealed that *LysMCre-Fasn*^*f/f*^ mice had increased infiltration of *S. aureus* in the airway (Fig. 6B). Furthermore, *LysMCre-Fasn*^*f/f*^ mice displayed a significant increase in the number of total cells and neutrophils in airway upon *S. aureus* infection (Fig. 6C, D). Cytokines were crucial proinflammatory molecules involved in the pathogenesis of *S. aureus* pneumonia. In *LysMCre-Fasn*^*f/f*^ mice, cytokines of CXCL1 and IL6 were significantly increased after *S. aureus* exposure as well (Fig. 6E, F). H&E staining of lung slices exhibited accumulation of inflammatory cells in peribronchial and perivascular regions of *LysMCre-Fasn*^*f/f*^ mice (Fig. 6G). Blinded scoring of these lung sections showed that the *LysMCre-Fasn*^*f/f*^ mice were more inflamed than the *Fasn*^*f/f*^ mice (Fig. 6H). This upregulation of inflammation, along with the increased bacterial burden, is consistent with the function of FASN in protecting the host from inflammation. In line with the above results, under severe infection conditions, *LysMCre-Fasn*^*f/f*^ mice were more susceptibility to lethal pneumonia. Survival analysis using Kaplan-Meier survival curves showed that FASN-deficient mice had significantly reduced survival (Fig. 6I). These findings establish the fact that FASN is fundamental for macrophage’s immune response during *S. aureus* infection. FASN deficiency leads to the impaired bacterial clearance of macrophages and aggravates *S. aureus* pneumonia.

## Discussion

*S. aureus* has evolved multiple mechanisms to counteract macrophages during infection. In the studies detailed in this article, we demonstrated that FASN plays a crucial role in macrophage-mediated defense against *S. aureus* infection. FASN, known for its role in de novo fatty acid synthesis, was significantly upregulated in macrophages following *S. aureus* infection. This upregulation led to an increase in LD formation, which facilitated enhanced bacterial killing without affecting macrophage phagocytic capacity. Importantly, FASN deficiency in macrophages resulted in impaired bacterial clearance and worsened outcomes in a murine model of *S. aureus* pneumonia. These findings highlight the role of FASN in coordinating metabolic and immune responses in macrophages during bacterial infection.

Immunometabolism has garnered significant attention in recent years due to its pivotal role in regulating immune responses and shaping the fate of immune cells. FASN-mediated endogenous fatty acid synthesis is a critical component in this regulatory network, influencing various aspects of immune cell function [34, 35]. Emerging evidence highlights that FASN-generated fatty acids modulate membrane composition and impact key signaling pathways such as Rho GTPase trafficking, which is essential for macrophage adhesion, migration, and activation in metabolic disorders like diabetes [36]. Fatty acid synthesis is also required for M-CSF dependent differentiation of primary human monocytes [37]. Despite these advancements, the role of de novo lipogenesis in macrophage-mediated killing of pathogens remains underexplored. Our study fills this gap by demonstrating that FASN expression is significantly upregulated during *S. aureus* infection, underscoring its importance in macrophage antimicrobial responses. Through a combination of genetic and pharmacological inhibition of FASN, we show that de novo fatty acid synthesis is indispensable for macrophage-mediated bacterial clearance. This role of FASN suggests a broader immunometabolic framework where lipid metabolism is a key determinant of immune responses, and manipulating this pathway could offer therapeutic potential.

To be more detailed, we identified that the proteasome activity was hindered in *S. aureus*-infected macrophages. It has been well known that UPS is a major proteolytic system that controls protein degradation [26]. The loss of proteasomes would accumulate the ubiquitinated proteins. Indeed, we observed an increase in the levels of ubiquitinated FASN protein in macrophages following *S. aureus* infection. These findings suggest that *S. aureus* can functionally interact with the proteasome machinery to impair FASN degradation, leading to an elevated FASN level in infected macrophages. Our subsequent investigations revealed that this regulation of proteasome activity by *S. aureus* is mediated through the PI3K/AKT/mTOR signaling pathway. The PI3K/AKT/mTOR axis is a well-established pathway involved in cellular metabolism, proliferation, and survival, and has recently been implicated in the regulation of proteasome function [27]. In our study, *S. aureus* infection activated the PI3K/AKT/mTOR pathway and suppressed the activation of proteasome. The inhibition of mTOR restored proteasome activity and facilitated the degradation of FASN, further confirming the critical role of the PI3K/AKT/mTOR pathway in this process. This interaction between *S. aureus* and the PI3K/AKT/mTOR pathway represents an important intersection of immune regulation and metabolic control. The ability of *S. aureus* to suppress proteasome activity through this pathway not only enhances FASN levels but also modulates LD formation, which plays an important role in host defense. As such, targeting the PI3K/AKT/mTOR pathway to enhance macrophage bactericidal function could be a promising therapeutic strategy for controlling *S. aureus* infections.

How does macrophage FASN exert its function on antimicrobial response to *S. aureus* infection? FASN is a key enzymatic complex in lipogenesis by catalyzing acetyl-CoA and malonyl-CoA to palmitic acid, which governs the synthesis of long-chain fatty acids (FAs) [12]. LDs are considered as the major lipid storage organelles of eukaryotic cells [17]. Our data suggested that the number of LDs in *S. aureus*-treated macrophages was significantly increased. Furthermore, our findings indicated that the LD formation was dependent on FASN since inhibition of FASN could directly suppress the accumulation of LDs in *S. aureus*-infected macrophages. A previous report showed that pathogens require host-derived lipids provided by LDs to support their survival [38]. As a result, LDs also have the potential to deliver host defenses against infected pathogens [18]. In line with this, our data suggested that LD loading was beneficial for the antibacterial response of the macrophages against *S. aureus*. Notably, LDs isolated from infected macrophages were enriched in the antimicrobial peptide CAMP. This aligns with findings from Bosch et al. [18], who demonstrated that LD-localized CAMP enhances host defense against multiple bacterial species, including E. coli, Listeria monocytogenes, and MRSA. Notably, their study showed that silencing CAMP abolished the antibacterial effect of LDs, whereas overexpression of LD-resident CAMP significantly reduced bacterial burden in host cells. Building on this, our data further support the notion that FASN-dependent LD formation facilitates antibacterial responses, in part by recruiting CAMP. Consequently, our study demonstrates that FASN and LD formation are integral to the antimicrobial response of macrophages during *S. aureus* infection. By elucidating the mechanisms of FASN-dependent lipogenesis and its impact on macrophage function, we provide a foundation for future research aimed at targeting metabolic pathways in immune cells to enhance bacterial clearance and improve infection outcomes.

Our study demonstrates that *S. aureus* manipulates host immune responses by suppressing proteasome activity through the PI3K/AKT/mTOR pathway, which leads to impaired FASN degradation and subsequent LD accumulation in macrophages. These findings highlight the potential clinical value of targeting this pathway to enhance immune function and bacterial clearance. Future therapeutic strategies could involve liposome-based drug delivery systems. Liposomes, which are spherical vesicles composed of lipid bilayers [39], can be employed to encapsulate antibiotics, enhancing their delivery to infected tissues while minimizing systemic toxicity. By leveraging liposome-encapsulated antibiotics, we can exploit the natural lipid metabolism pathways of macrophages. FASN-mediated LDs could serve as an intracellular reservoir, releasing antibiotics in a controlled manner to enhance the local antimicrobial response. This approach has the potential to improve antibiotic penetration into infected macrophages, overcoming the challenges posed by intracellular pathogens such as *S. aureus*. Additionally, liposomes can be engineered to carry mTOR agonist or other modulators of the PI3K/AKT/mTOR pathway, delivering dual-function therapy that both inhibits proteasome activity and enhances the bactericidal capacity of macrophages. The combination of antibiotic-laden liposomes with immune-modulating agents could offer a powerful therapeutic strategy, particularly in patients with chronic infections or those who are at risk of antibiotic resistance.

## Conclusion

Taken together, our results presented here link the FASN-mediated de novo fatty acid synthesis to a macrophage-intrinsic host defense mechanism against *S. aureus*. We uncovered that *S. aureus* suppresses proteasome activity via the PI3K/AKT/mTOR pathway, contributing to the stabilization and accumulation of FASN. This process enhances LD formation, which plays a critical role in macrophage bactericidal activity. Inhibition of FASN leads to a reduction in LD formation, decreased bacterial clearance, and increased inflammation. Our findings establish a strong link between metabolic regulation and immune defense, highlighting the therapeutic potential of modulating FASN-mediated lipogenesis to boost immune defense and improve infection outcomes in *S. aureus* pneumonia.

## Supplementary information

Supplementary file

Supplementary Figure 1

Supplementary Figure 2

Supplementary Figure 3

Supplementary table

Original western blots

## Data Availability

All data needed to evaluate the conclusions in the paper are present in the paper and/or the Supplementary Materials. Proteomic data are publicly available via the iProX repository (accession no. PXD064032). Additional data related to this paper may be requested from the corresponding author upon reasonable request.
