# Supplementary material for: Fatty acid synthase-mediated lipid droplet formation enhances macrophage killing of Staphylococcus aureus
Source: Cell Death Dis. 2025 Oct 7;16(1):715. doi: 10.1038/s41419-025-08044-7 (PMC12504578; doi:10.1038/s41419-025-08044-7)
Supplement: Supplementary file 1 — Supplementary file [file 41419_2025_8044_MOESM1_ESM.docx]

**Fatty Acid Synthase-Mediated Lipid Droplet Formation Enhances Macrophage Killing of *Staphylococcus Aureus***

Yanping Wu^1#^, Jiaxin Shen^1#^, Shenwei Gao^1#^, Miao Li^1^, Qingyu Weng^1^, Kua Zheng^1^, Chen Zhu^1^, Zhongnan Qin^1^, Jieyu Li^1^, Jiafei Lou^1^, Songmin Ying^1^, Yinfang Wu^1^*, Zhihua Chen^1^*, Wen Li^1^*

^1^Key Laboratory of Respiratory Disease of Zhejiang Province, Department of Respiratory and Critical Care Medicine, Second Affiliated Hospital of Zhejiang University School of Medicine, Hangzhou, Zhejiang, 310009, China.

**Authorship note:** ^#^These authors contributed equally to this article.

***Corresponding authors:**

Wen Li, Email: liwen@zju.edu.cn, or Zhihua Chen, E-mail: zhihuachen@zju.edu.cn, or Yinfang Wu, Email: yinfangwu@zju.edu.cn.

Department of Respiratory and Critical Care Medicine, Second Affiliated Hospital, Zhejiang University School of Medicine, 88 Jiefang Rd, Hangzhou 310009, China. Tel.: 86-571-8778-3729; Fax: 86-571-8706-8001.

**Supplementary figure and table legends**

**Figure S1** Western blot analysis of FASN in BMDMs infected with *S. aureus* (MOI=10) for 9 hours and treated with MG132 (100 nM), rapamycin (25 nM), or both.

**Figure S2** Impact of FASN inhibition on macrophage activation, migration, and adhesion in response to *S. aureus* infection. (A, B) Macrophage migration was evaluated after treatment with 25 µM C75. Representative images of the scratch wound healing assay at 0 and 24 hours post-scratch (A) and quantitative analysis of scratch length (B). Scale bar, 200 μm. (C, D) Macrophage adhesion was evaluated after treatment with 25 µM C75 in THP-1 cells. Representative images of macrophages stained with crystal violet (C) and Quantitative analysis of the number of adherent macrophages (D). Scale bar, 200 μm. (E, F) qPCR analysis of *Il6* and *Il1β* mRNA in BMDM treated with *S. aureus* for 9 hours, in the presence or absence of C75 at the indicated concentrations. (G, H) BMDM isolated from *Fasn^f/f^* and *LysMCre-Fasn^f/f^* mice were cultured with *S. aureus* for 9 hours. qPCR analysis of *Il6* and *Il1β* mRNA was performed. All data are shown as mean ± SEM, analyzed using an unpaired two-tailed Student's t-test (D) or two-way ANOVA (B, E, F, G, H). **P* < 0.05, ***P* < 0.01, ****P* < 0.001, *****P* < 0.0001.

**Figure S3** Impact of FASN knockdown and lipogenic enzyme inhibition on LD formation and antibacterial response. (A) BMDM were transfected with control or FASN siRNA and infected with *S. aureus* (MOI=10) for 9 hours. LD formation was assessed by BODIPY 493/503 staining, and bacterial burden was evaluated by CFU assay. Green, BODIPY 493/503; blue, DAPI. Scale bar, 20 μm. (B) Quantification of the number of LDs per cell. (C) Measurement of bacterial colony number. (D, E) Knockdown efficiency of FASN in BMDM transfected with FASN siRNA. FASN mRNA level was measured by qPCR (D), and FASN protein level was analyzed by Western blot (E). (F) BMDM were treated with ACC inhibitor (CP-640186, 20 µM), ACLY inhibitor (BMS-303141, 20 µM), DGAT1 inhibitor (A922500, 20 µM), or DGAT2 inhibitor (PF-06424439, 20 µM), and infected with *S. aureus* (MOI=10) for 9 hours. LD formation was assessed by BODIPY 493/503 staining, and bacterial burden was evaluated by CFU assay. Green, BODIPY 493/503; blue, DAPI. Scale bar, 20 μm. (G) Quantification of the number of LDs per cell. (H) Measurement of bacterial colony number. All data are shown as mean ± SEM and analyzed using an unpaired two-tailed Student's t-test. **P* < 0.05, ***P* < 0.01, ****P* < 0.001, *****P* < 0.0001.

**Supplementary table 1** Mean differences, 95% confidence intervals (CI) of difference and adjusted *P* values for compared groups.
