# Supplementary figures and images for: Fatty acid synthase-mediated lipid droplet formation enhances macrophage killing of Staphylococcus aureus
Source: Cell Death Dis. 2025 Oct 7;16(1):715. doi: 10.1038/s41419-025-08044-7 (PMC12504578; doi:10.1038/s41419-025-08044-7)

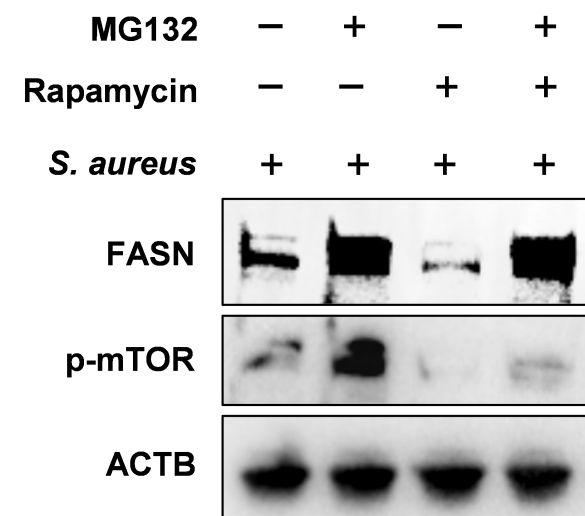

Supplement: Supplementary file 2 — Supplementary Figure 1 [file 41419_2025_8044_MOESM2_ESM.pdf]

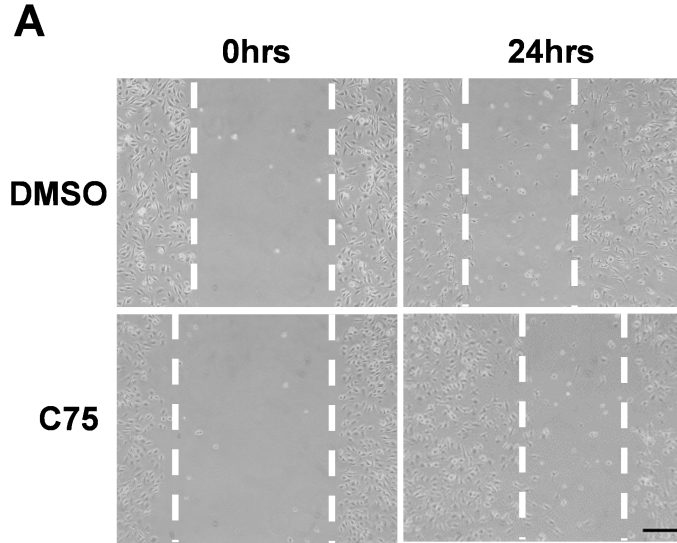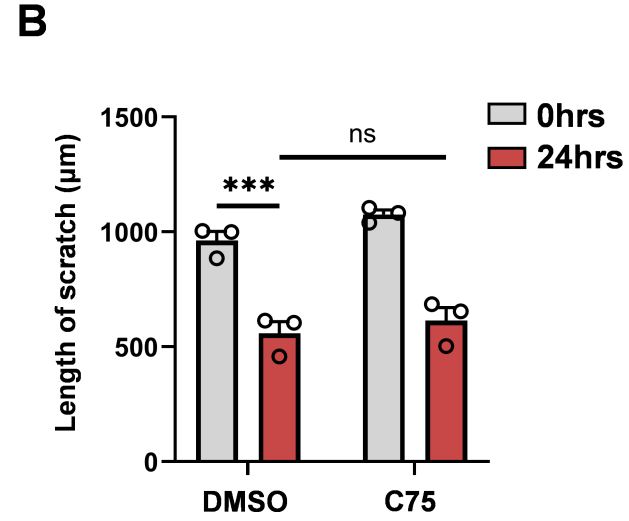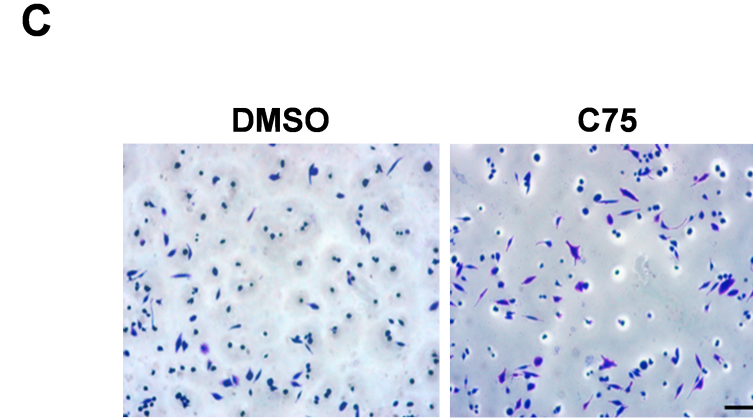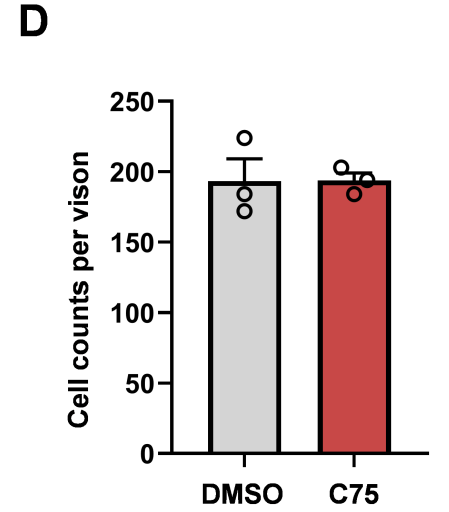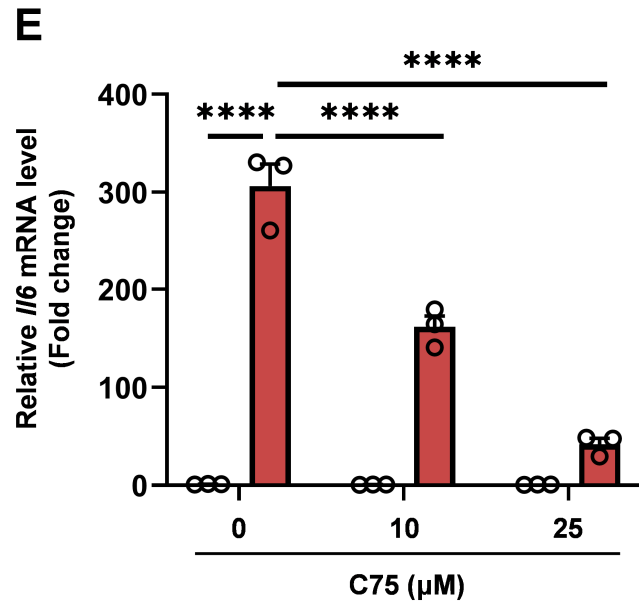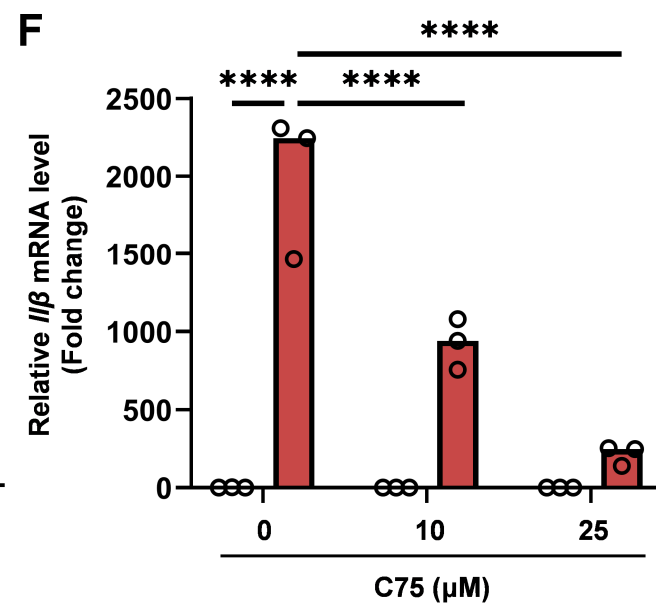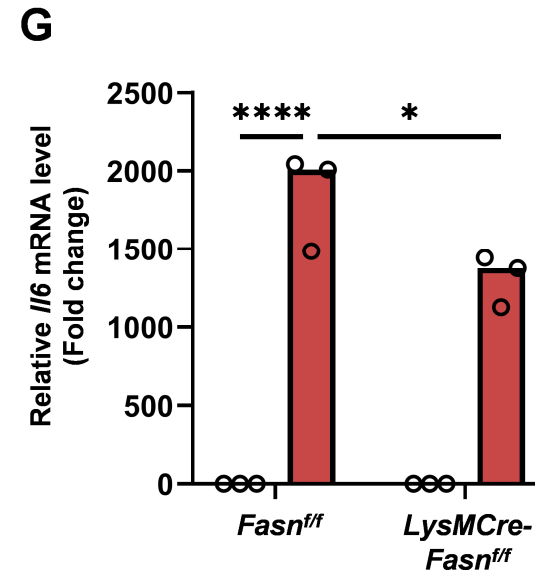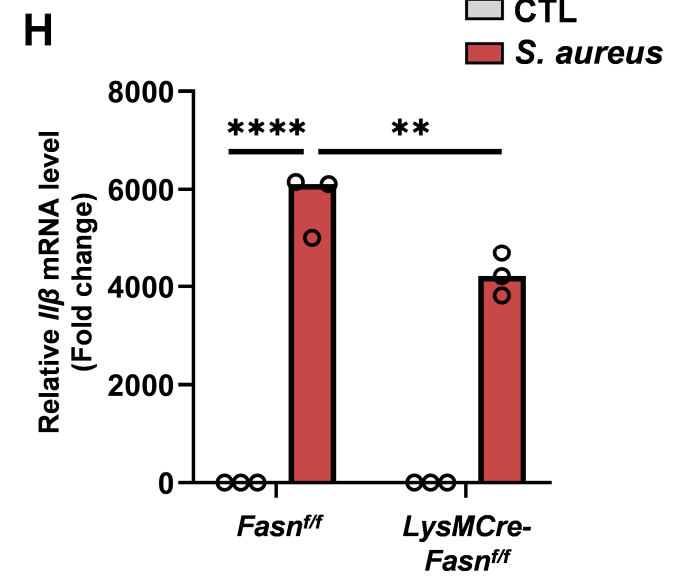

Supplement: Supplementary file 3 — Supplementary Figure 2 [file 41419_2025_8044_MOESM3_ESM.pdf]

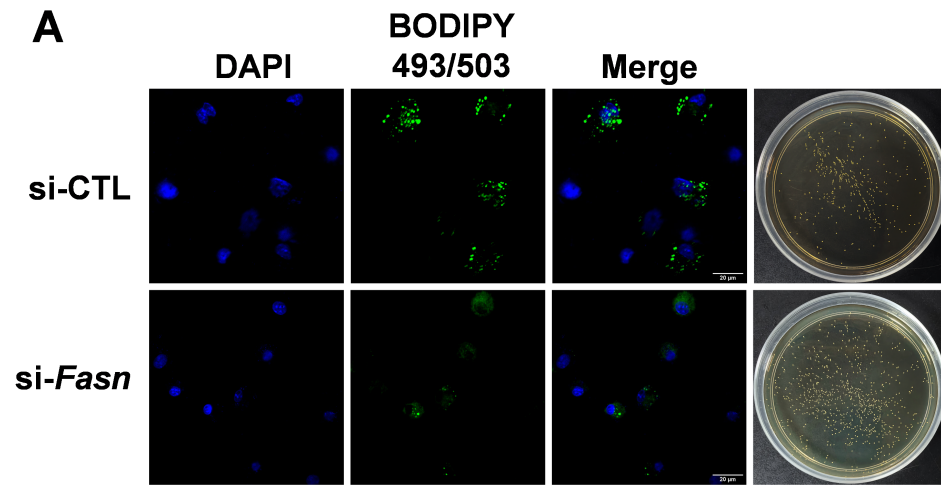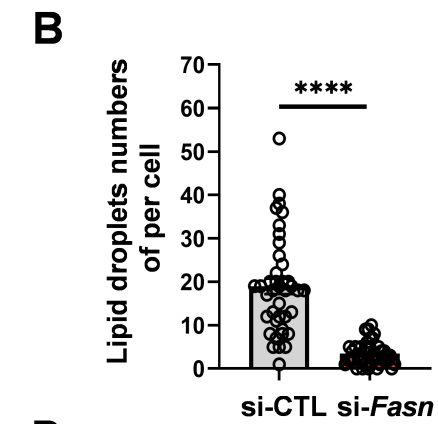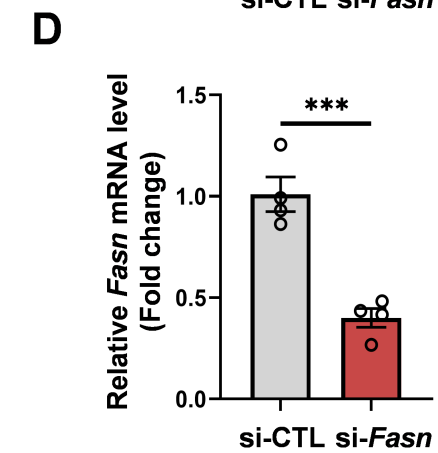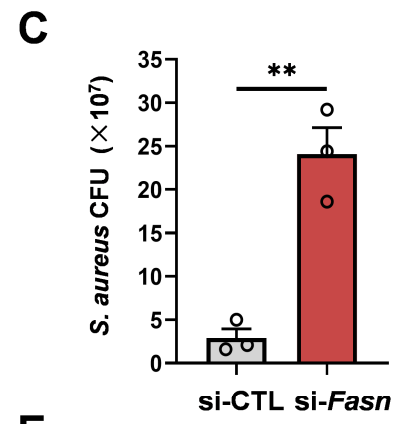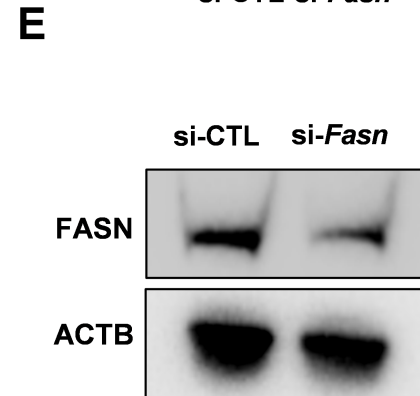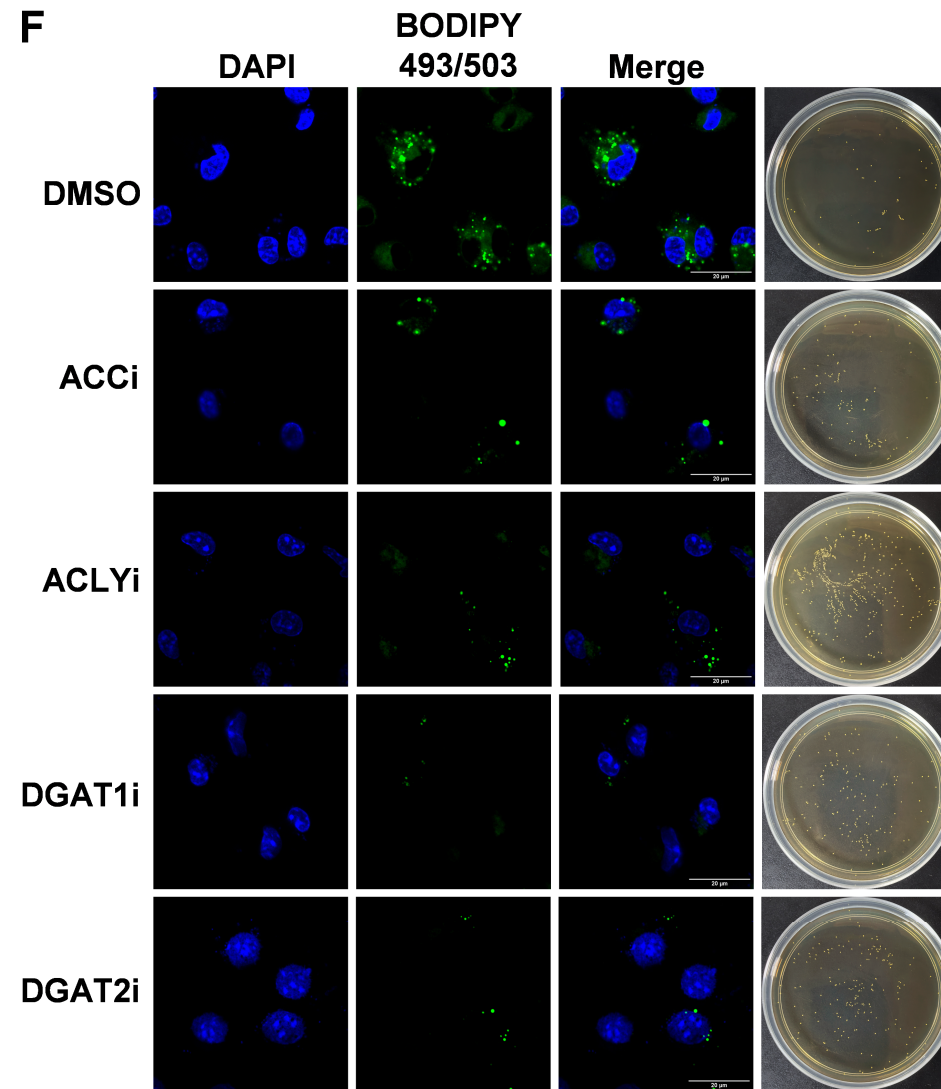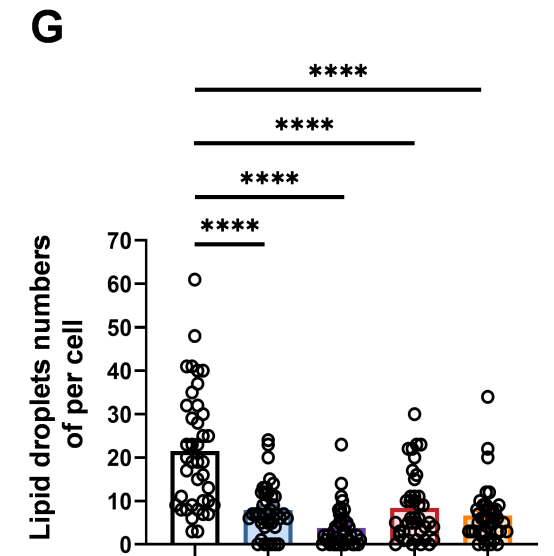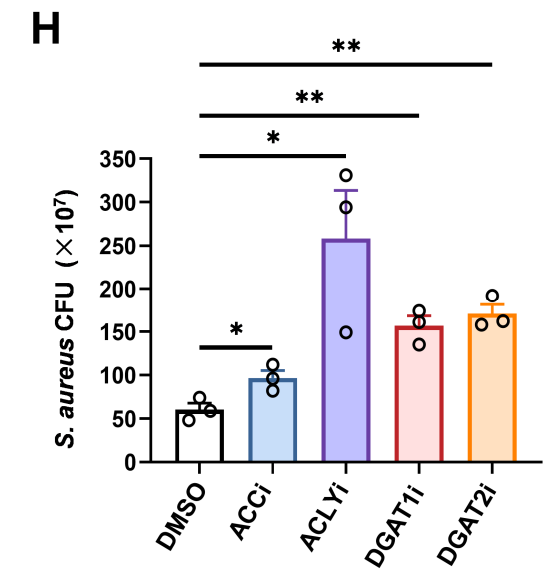

Supplement: Supplementary file 4 — Supplementary Figure 3 [file 41419_2025_8044_MOESM4_ESM.pdf]
