# Supplementary material for: Fatty acid synthase-mediated lipid droplet formation enhances macrophage killing of Staphylococcus aureus
Source: Cell Death Dis. 2025 Oct 7;16(1):715. doi: 10.1038/s41419-025-08044-7 (PMC12504578; doi:10.1038/s41419-025-08044-7)
Supplement: Supplementary file 5 — Supplementary table [file 41419_2025_8044_MOESM5_ESM.docx]

**Supplementary table 1.** Mean differences, 95% confidence intervals (CI) of difference and adjusted *P* values for compared groups

| **Figures** | **Compared groups** | **Mean difference** | **95% CI of difference** | ***P* Value** |
| --- | --- | --- | --- | --- |
| Figure 1E | CTL vs *S. aureus* | 0.6256 | 0.1827 to 1.069 | 0.0172 |
| Figure 1G | CTL vs *S. aureus* | 0.9283 | 0.1259 to 1.731 | 0.0325 |
| Figure 2A | *S. aureus* (0 vs 6 hrs) | 0.4726 | 0.2342 to 0.7109 | 0.0010 |
|  | *S. aureus* (0 vs 9 hrs) | 0.7370 | 0.4987 to 0.9754 | <0.0001 |
| Figure 2B | CTL vs *S. aureus* | -0.8639 | -0.9458 to -0.7820 | <0.0001 |
| Figure 2G | DMSO (CTL vs *S. aureus*) | 0.7362 | 0.3412 to 1.131 | 0.0018 |
|  | *S. aureus* (DMSO vs Rapamycin) | -0.4977 | -0.8927 to -0.1027 | 0.0172 |
| Figure 3B | *S. aureus* (DMSO vs C75) | 26.56 | 13.92 to 39.20 | 0.0013 |
| Figure 3D | *S. aureus*  (*Fasn^f/f^* vs *LysMCre-Fasn^f/f^* ) | 62.00 | 38.51 to 85.49 | 0.0018 |
| Figure 3F | *S. aureus*  (*Fasn^f/f^* vs *LysMCre-Fasn^f/f^* ) | 5.600 | 2.091 to 9.109 | 0.0035 |
| Figure 3H | *S. aureus*  (*Fasn^f/f^* vs *LysMCre-Fasn^f/f^* ) | 1.507 | -0.6877 to 3.701 | 0.1293 |
| Figure 4B | *S. aureus* (0 vs 9 hrs) | -0.5103 | -0.8896 to -0.1311 | 0.0111 |
|  | *S. aureus* (0 vs 12 hrs) | -0.6586 | -1.038 to -0.2793 | 0.0024 |
| Figure 4D | CTL vs *S. aureus* | 23.13 | 19.20 to 27.05 | <0.0001 |
| Figure 4F | CTL vs *S. aureus* | 10.65 | 9.569 to 11.73 | <0.0001 |
| Figure 5C | *S. aureus* vs *S. aureus+*C75 | 0.6141 | 0.1077 to 1.121 | 0.0208 |
| Figure 5F | *S. aureus* vs *S. aureus*+OA | -2.963 | -4.049 to -1.877 | 0.0001 |
| Figure 5H | *S. aureus* (CTL vs LDs) | -36.67 | -51.10 to -22.23 | 0.0021 |
| Figure 5J | *S. aureus* (CTL vs OA) | -8.803 | -11.82 to -5.787 | 0.0013 |
| Figure 6A | *S. aureus*  (*Fasn^f/f^* vs *LysMCre-Fasn^f/f^* ) | 2.551 | 0.8732 to 4.230 | 0.0074 |
| Figure 6C | *Fasn^f/f^* (CTL vs *S. aureus*) | -4.630 | -7.765 to -1.495 | 0.0044 |
|  | *S. aureus*  (*Fasn^f/f^* vs *LysMCre-Fasn^f/f^* ) | -5.292 | -8.427 to -2.157 | 0.0015 |
| Figure 6D | *Fasn^f/f^* (CTL vs *S. aureus*) | -5.392 | -7.799 to -2.986 | <0.0001 |
|  | *S. aureus*  (*Fasn^f/f^* vs *LysMCre-Fasn^f/f^* ) | -5.609 | -8.015 to -3.203 | <0.0001 |
| Figure 6E | *Fasn^f/f^* (CTL vs *S. aureus*) | -26.33 | -44.48 to -8.177 | 0.0050 |
|  | *S. aureus*  (*Fasn^f/f^* vs *LysMCre-Fasn^f/f^* ) | -21.39 | -39.55 to -3.241 | 0.0205 |
| Figure 6F | *S. aureus*  (*Fasn^f/f^* vs *LysMCre-Fasn^f/f^* ) | -97.97 | -197.0 to 1.098 | 0.0528 |
| Figure 6H | *Fasn^f/f^* (CTL vs *S. aureus*) | -1.400 | -2.180 to -0.6200 | 0.0008 |
|  | *S. aureus*  (*Fasn^f/f^* vs *LysMCre-Fasn^f/f^* ) | -0.8000 | -1.580 to -0.02002 | 0.0441 |
| Figure 6I | *S. aureus*  (*Fasn^f/f^* vs *LysMCre-Fasn^f/f^* ) | / | / | 0.0095 |
| Supplementary figure 2B | DMSO (0hrs vs 24 hrs) | 404.2 | 235.2 to 573.2 | 0.0004 |
|  | 24 hrs (DMSO vs C75) | -55.37 | -224.4 to 113.7 | 0.6340 |
| Supplementary figure 2D | DMSO vs C75 | 0.3333 | -45.89 to 46.56 | 0.9850 |
| Supplementary figure 2E (*Il6*) | CTL vs *S. aureus* | -305.2 | -346.8 to -263.5 | <0.0001 |
|  | *S. aureus*  (C75 0μM vs C75 10μM ) | 144.8 | 104.6 to 184.9 | <0.0001 |
|  | *S. aureus*  (C75 0μM vs C75 25μM ) | 264.4 | 224.3 to 304.6 | <0.0001 |
| Supplementary figure 2F (*Il1β*) | CTL vs *S. aureus* | -2005 | -2468 to -1541 | <0.0001 |
|  | *S. aureus*  (C75 0μM vs C75 10μM ) | 1081 | 634.2 to 1528 | <0.0001 |
|  | *S. aureus*  (C75 0μM vs C75 25μM ) | 1792 | 1346 to 2239 | <0.0001 |
| Supplementary figure 2G (*Il6*) | *Fasn^f/f^* (CTL vs *S. aureus*) | -1844 | -2241 to -1447 | <0.0001 |
|  | *S. aureus*  (*Fasn^f/f^* vs *LysMCre-Fasn^f/f^* ) | 531.2 | 134.1 to 928.3 | 0.0126 |
| Supplementary figure 2H (*Il1β*) | *Fasn^f/f^* (CTL vs *S. aureus*) | -5755 | -6629 to -4881 | <0.0001 |
|  | *S. aureus*  (*Fasn^f/f^* vs *LysMCre-Fasn^f/f^* ) | 1518 | 644.3 to 2392 | 0.0028 |
| Supplementary figure 3B | *S. aureus*  (si-CTL vs si-*Fasn* ) | -15.5 | -19.14 to -11.86 | <0.0001 |
| Supplementary figure 3C | *S. aureus*  (si-CTL vs si-*Fasn* ) | 21.17 | 12.16 to 30.17 | 0.0028 |
| Supplementary figure 3D | si-CTL vs si-*Fasn* | -0.6099 | -0.8483 to -0.3716 | 0.0008 |
| Supplementary figure 3G | *S. aureus*  (DMSO vs ACCi) | -13.50 | -18.24 to -8.765 | <0.0001 |
|  | *S. aureus*  (DMSO vs ACLYi) | -17.68 | -22.28 to -13.07 | <0.0001 |
|  | *S. aureus*  (DMSO vs DGAT1i) | -13.08 | -18.08 to -8.066 | <0.0001 |
|  | *S. aureus*  (DMSO vs DGAT2i) | -14.83 | -19.64 to -10.01 | <0.0001 |
| Supplementary figure 3H | *S. aureus*  (DMSO vs ACCi) | 36.33 | 4.448 to 68.22 | 0.0341 |
|  | *S. aureus*  (DMSO vs ACLYi) | 197.7 | 42.06 to 353.3 | 0.0243 |
|  | *S. aureus*  (DMSO vs DGAT1i) | 96.33 | 58.24 to 134.4 | 0.0022 |
|  | *S. aureus*  (DMSO vs DGAT2i) | 110.3 | 73.93 to 146.7 | 0.0011 |
