# Supplementary material for: Fatty acid synthase-mediated lipid droplet formation enhances macrophage killing of Staphylococcus aureus
Source: Cell Death Dis. 2025 Oct 7;16(1):715. doi: 10.1038/s41419-025-08044-7 (PMC12504578; doi:10.1038/s41419-025-08044-7)

Figure 1A

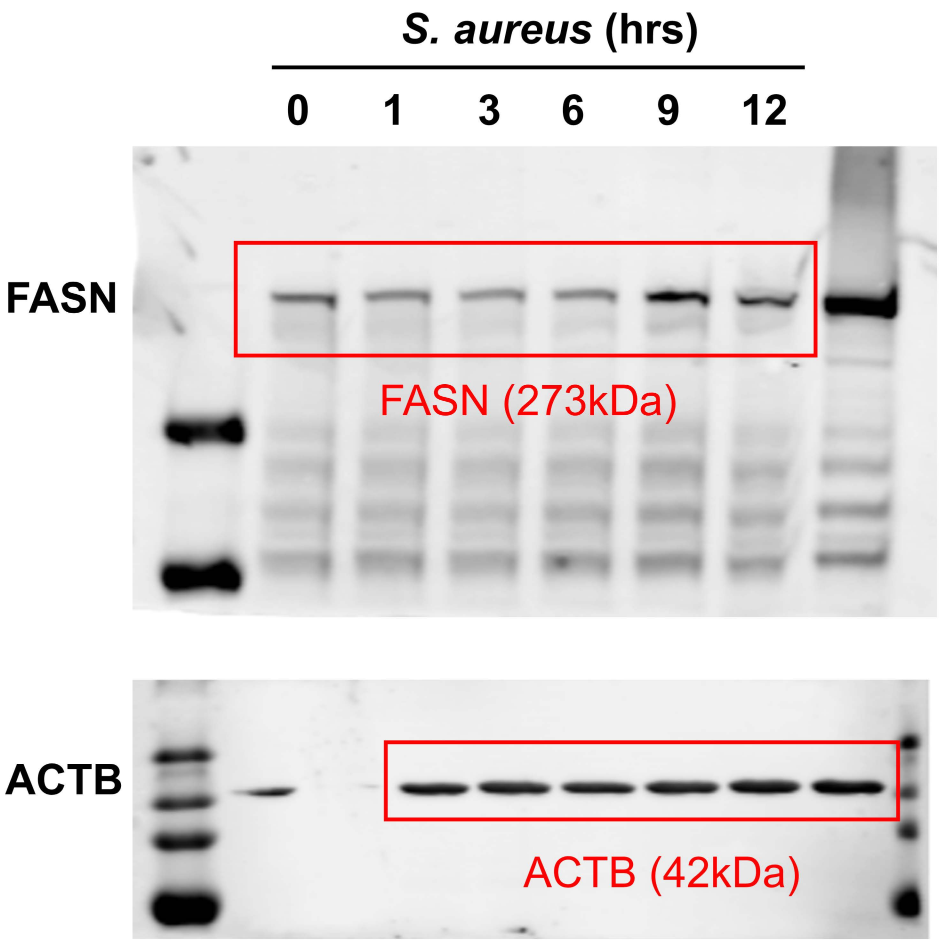

Figure 1B

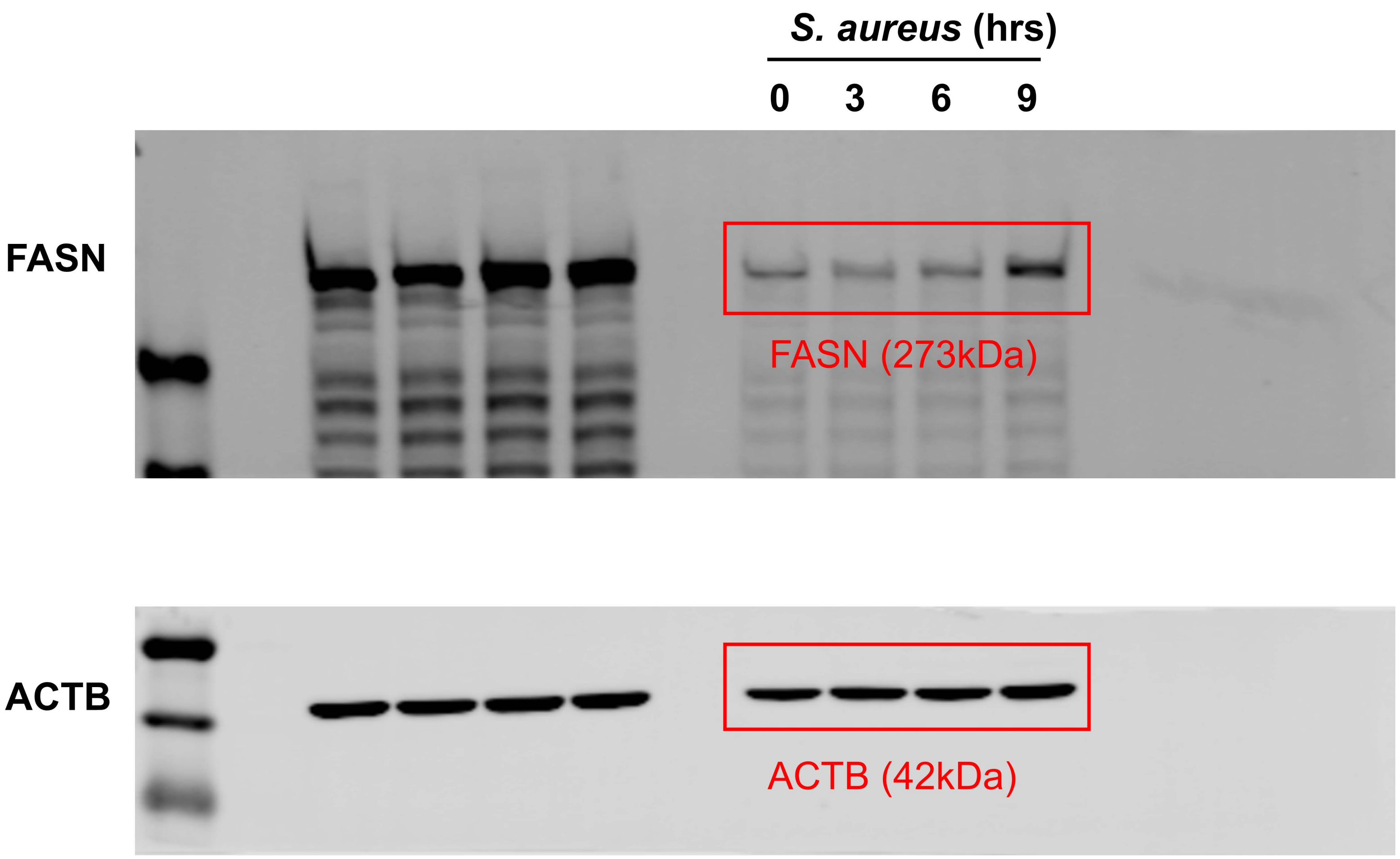

Figure 1C

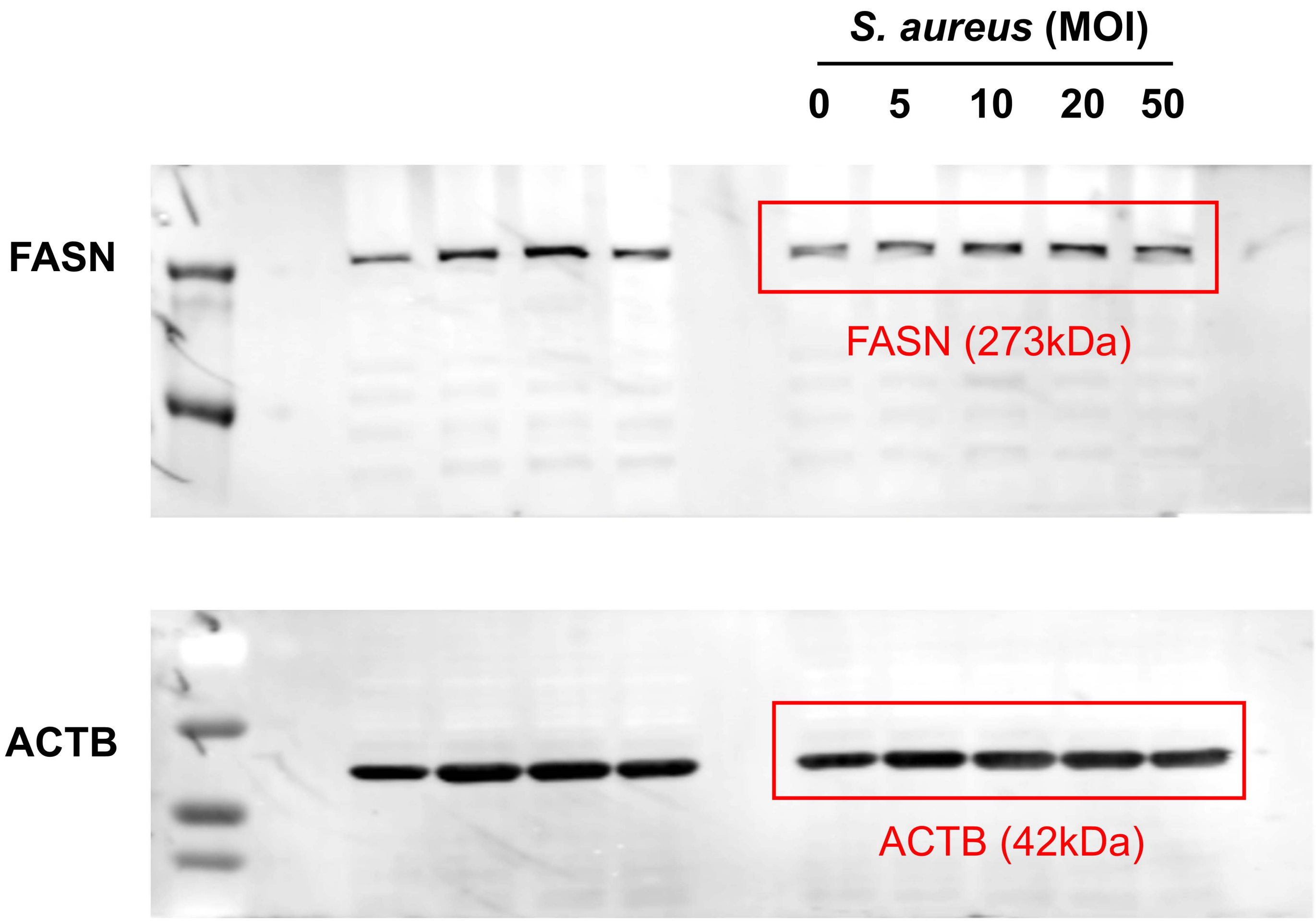

Figure 2C

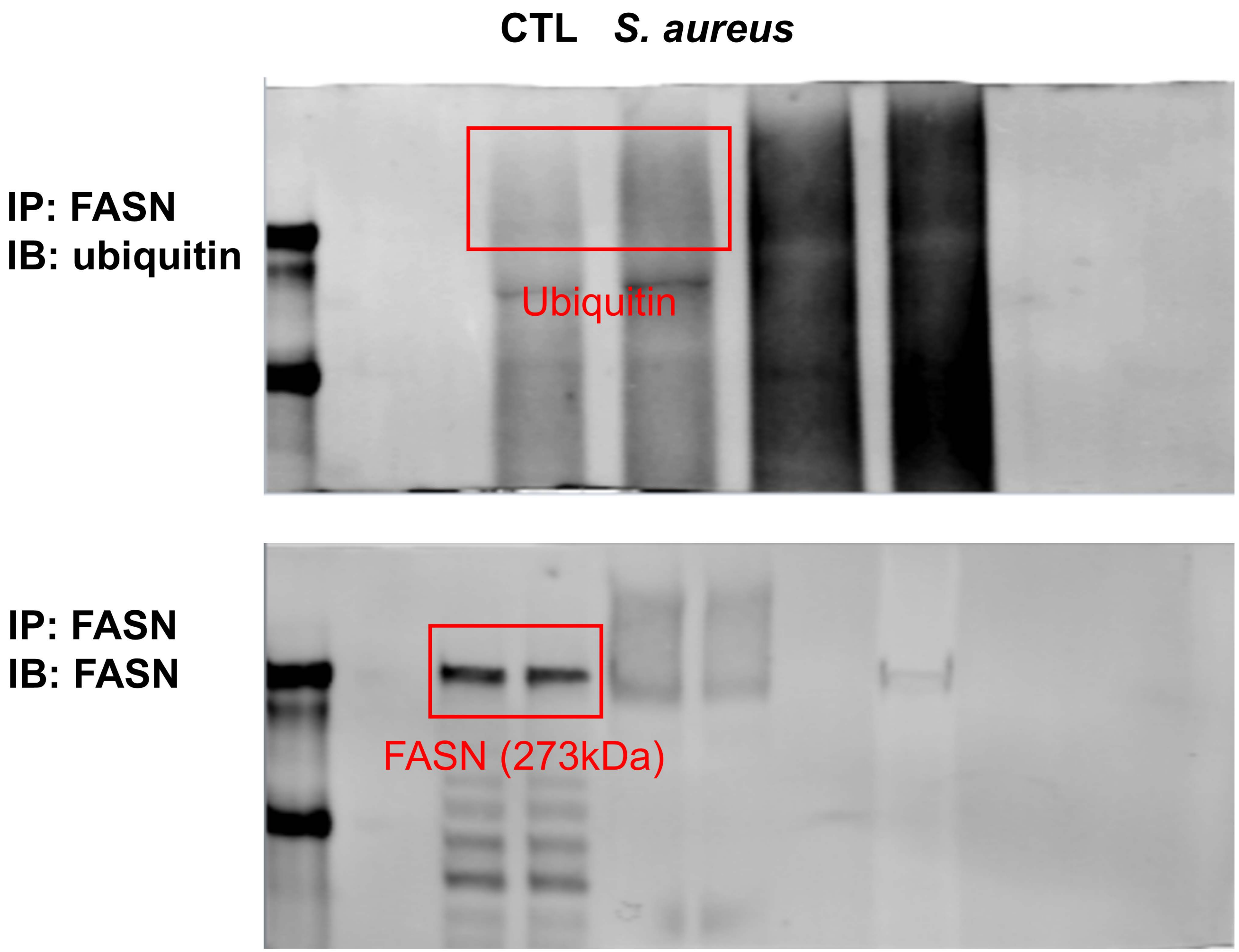

CTL
S. aureus
IP: FASN
IB: ubiquitin
Ubiquitin
IP: FASN
IB: FASN
FASN (273kDa)

Figure 2D

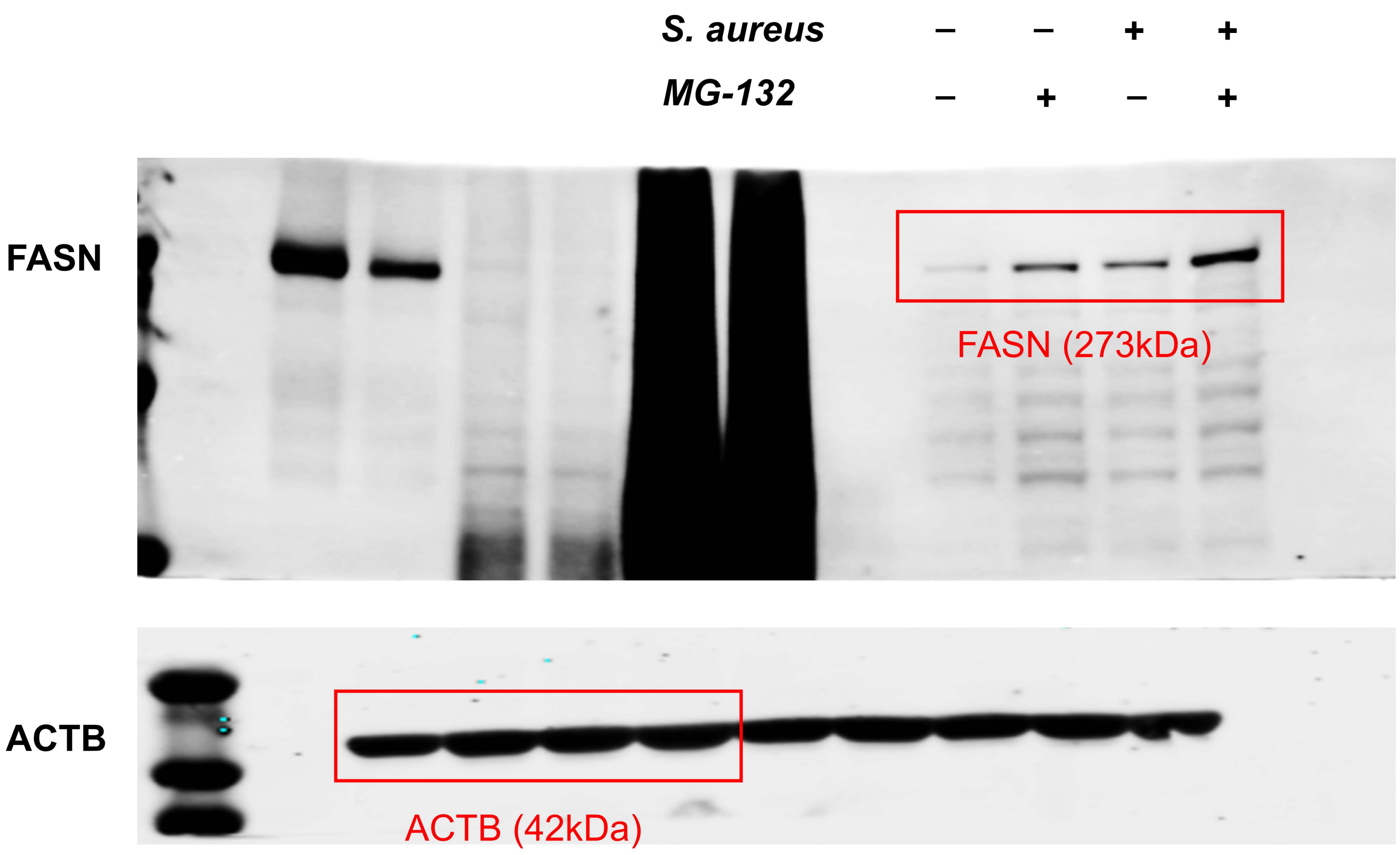

Figure 2F

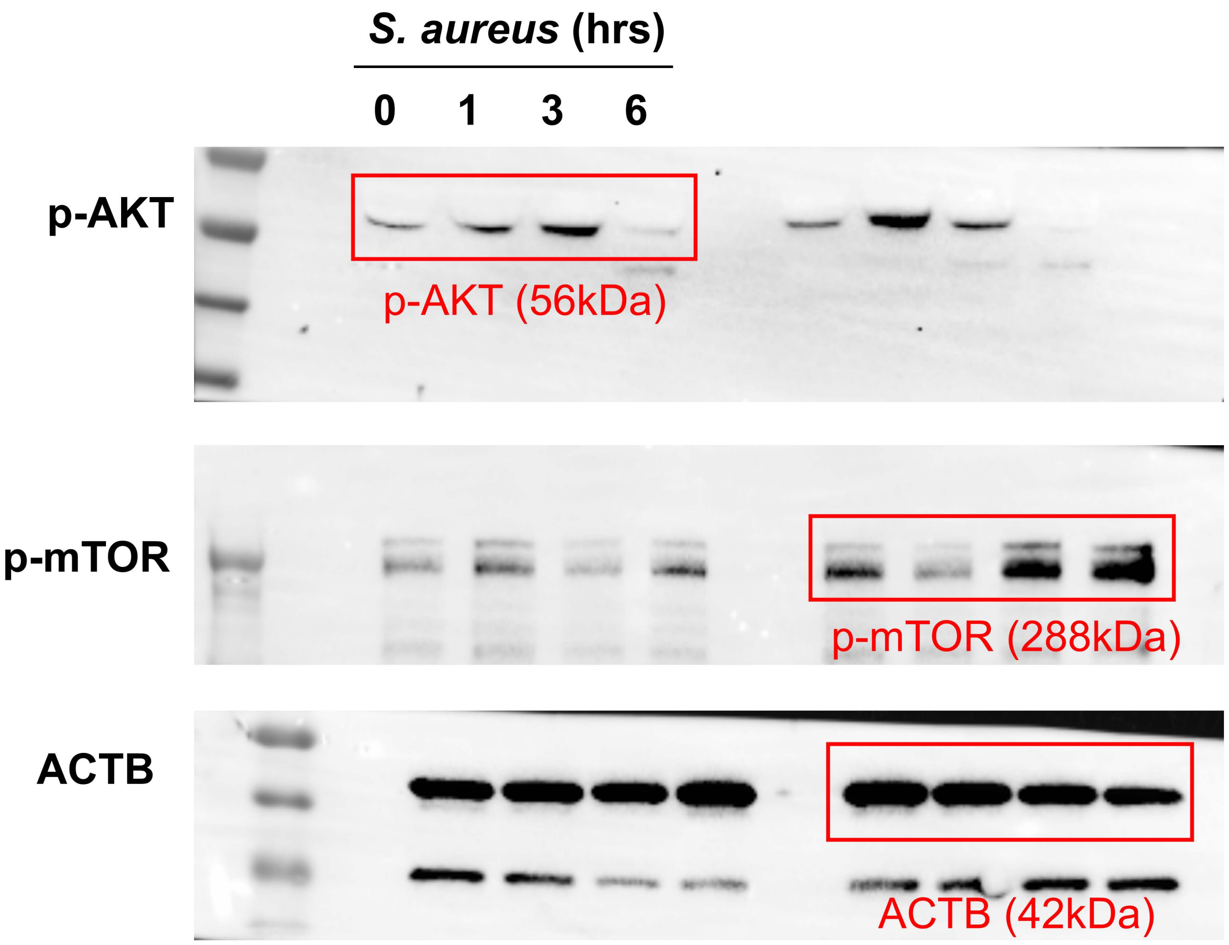

S. aureus (hrs)
0
1
3
6
p-AKT
p-AKT (56kDa)
p-mTOR
p-mTOR (288kDa)
ACTB
ACTB (42kDa)

Figure 2H

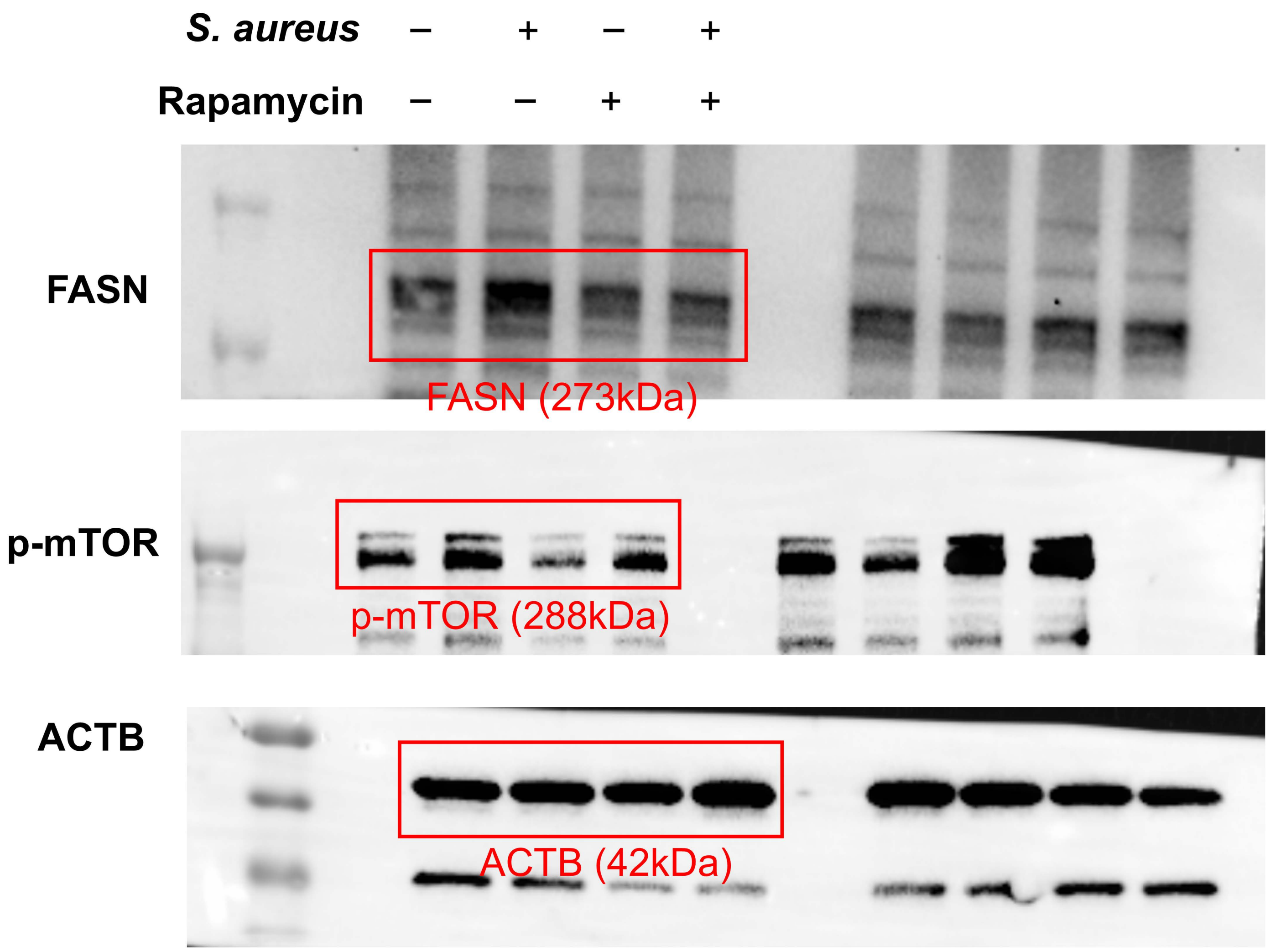

S. aureus – + – +
Rapamycin – – + +
FASN
FASN (273kDa)
p-mTOR
p-mTOR (288kDa)
ACTB
ACTB (42kDa)

Figure 5K

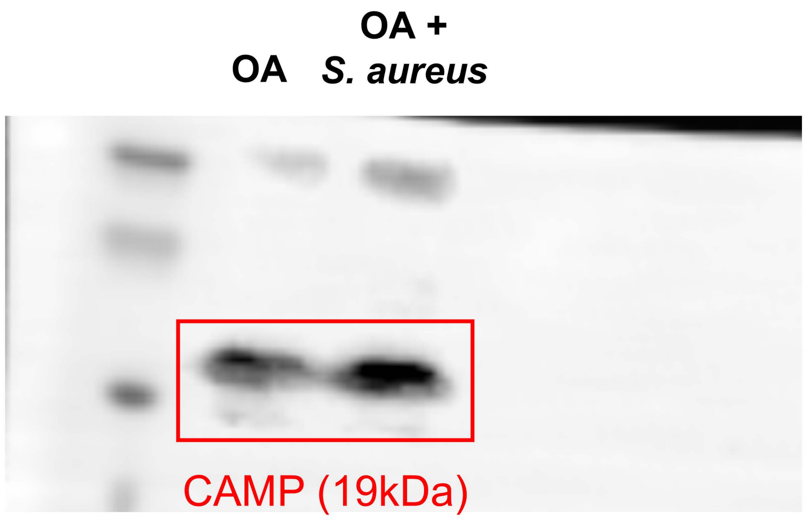

Supplementary Figure 1

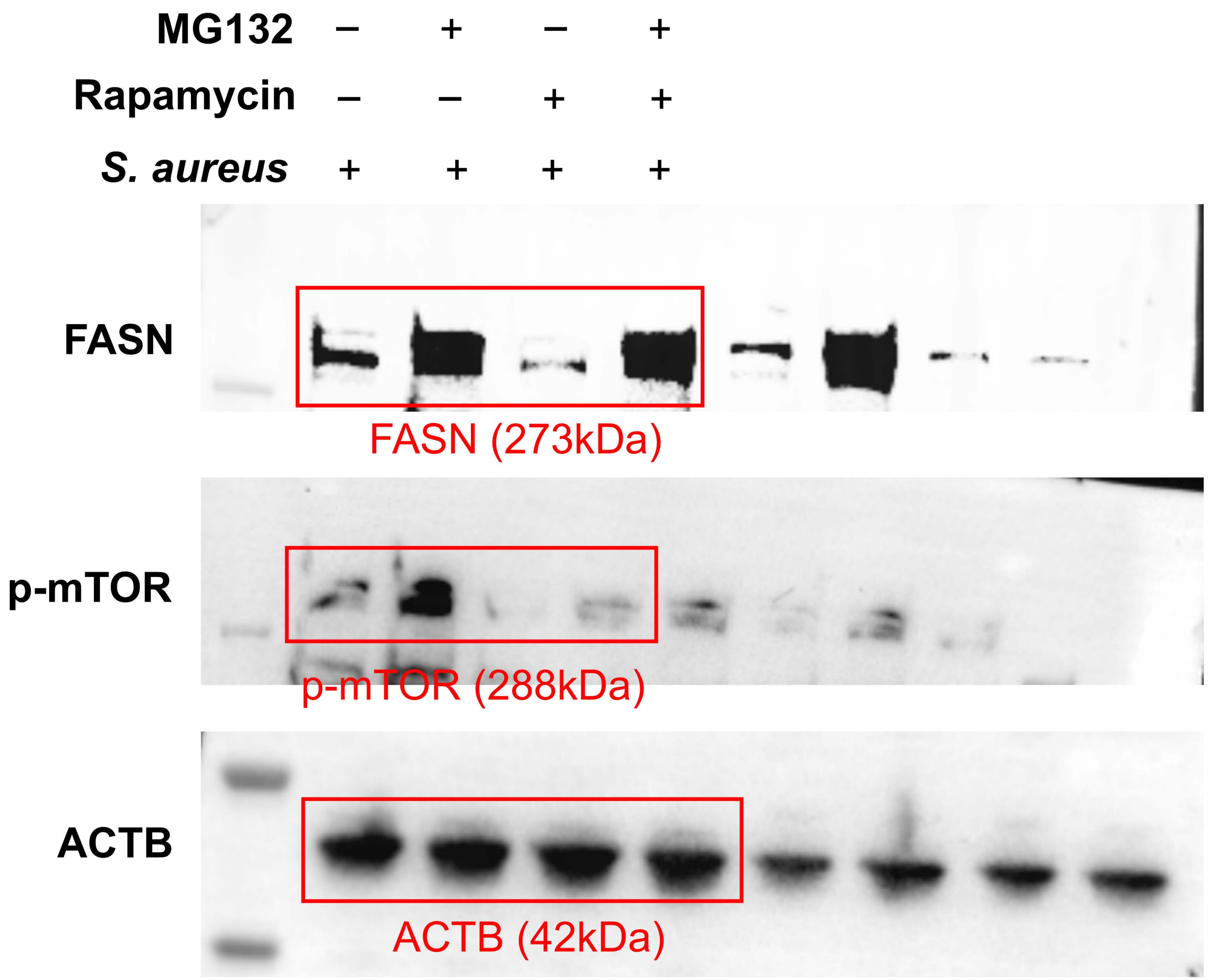

Supplementary Figure 3E

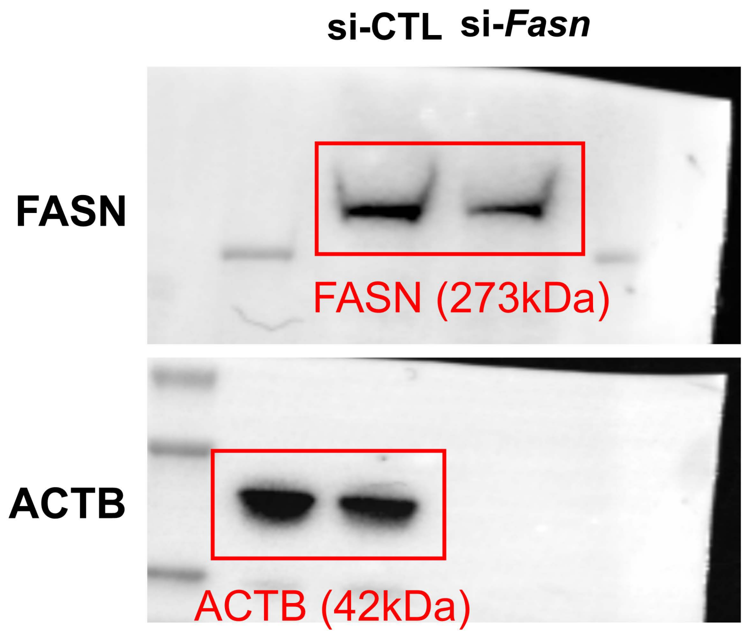

Supplement: Supplementary file 6 — Original western blots [file 41419_2025_8044_MOESM6_ESM.pdf]
